# Illuminating milling mechanochemistry by tandem real-time fluorescence emission and Raman spectroscopy monitoring†

**DOI:** 10.1039/d3sc04082h

**Published:** 2023-10-12

**Authors:** Patrick A. Julien, Mihails Arhangelskis, Luzia S. Germann, Martin Etter, Robert E. Dinnebier, Andrew J. Morris, Tomislav Friščić

**Affiliations:** a Department of Chemistry, McGill University 801 Sherbrooke St. W. H3A 0B8 Montreal Canada tomislav.friscic@mcgill.ca; b Department of Chemistry and Chemical Engineering, Royal Military College of Canada 13 General Crerar Crescent K7K 7B4 Kingston Canada patrick.julien@rmc-cmr.ca; c Faculty of Chemistry, University of Warsaw 1 Pasteura St. 02-093 Warsaw Poland m.arhangelskis@uw.edu.pl; d Max-Planck Institute for Solid State Research Heisenbergstrasse 1 D-70569 Stuttgart Germany; e Deutsches-Elektronen Synchrotron (DESY) Notkestrasse 85 22607 Hamburg Germany; f School of Metallurgy and Materials, University of Birmingham Birmingham B15 2TT UK a.j.morris.1@bham.ac.uk; g School of Chemistry, University of Birmingham Edgbaston Birmingham B15 2TT UK t.friscic@bham.ac.uk

## Abstract

In pursuit of accessible and interpretable methods for direct and real-time observation of mechanochemical reactions, we demonstrate a tandem spectroscopic method for monitoring of ball-milling transformations combining fluorescence emission and Raman spectroscopy, accompanied by high-level molecular and periodic density-functional theory (DFT) calculations, including periodic time-dependent (TD-DFT) modelling of solid-state fluorescence spectra. This proof-of-principle report presents this readily accessible dual-spectroscopy technique as capable of observing changes to the supramolecular structure of the model pharmaceutical system indometacin during mechanochemical polymorph transformation and cocrystallisation. The observed time-resolved *in situ* spectroscopic and kinetic data are supported by *ex situ* X-ray diffraction and solid-state nuclear magnetic resonance spectroscopy measurements. The application of first principles (*ab initio*) calculations enabled the elucidation of how changes in crystalline environment, that result from mechanochemical reactions, affect vibrational and electronic excited states of molecules. The herein explored interpretation of both real-time and *ex situ* spectroscopic data through *ab initio* calculations provides an entry into developing a detailed mechanistic understanding of mechanochemical milling processes and highlights the challenges of using real-time spectroscopy.

## Abstract 2

We demonstrate a tandem spectroscopic method for *in situ* monitoring of mechanochemical reactions by fluorescence emission and Raman spectroscopy, accompanied by periodic time-dependent density-functional theory (TD-DFT) modelling.

## Graphical abstract

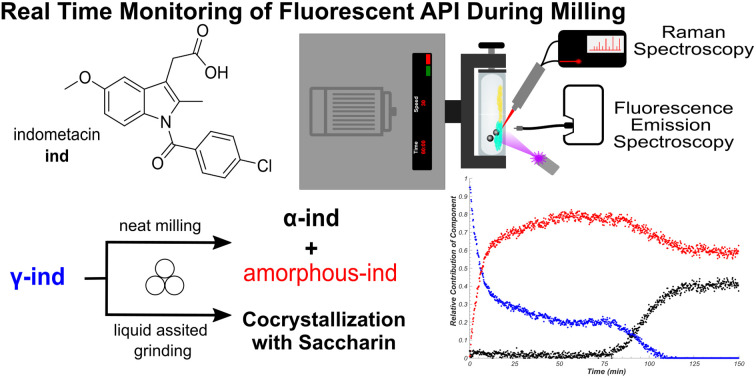

## Introduction

Mechanochemical reactions, driven or sustained by milling, grinding, or other types of mechanical agitation, have emerged as a uniquely general route to conduct chemical and materials synthesis without bulk solvents. The potential of mechanochemistry in sustainable synthesis was recognised in 2019 by International Union of Pure and Applied Chemistry (IUPAC), who placed it among the top ten emerging chemical technologies.^1,2^ Despite a wide range of existing, as well as nascent applications, the fundamental understanding of reactions under milling conditions remains limited. The lack of understanding of how chemical and materials transformations take place in the complex and highly dynamic ball-milling environment, involving shear, impact, and frictional heating, is an obstacle for further development of mechanochemical processes. Time-resolved *in situ* (TRIS)^3^ monitoring based on X-ray powder diffraction^4^ (XRPD) or Raman spectroscopy^5^ has recently emerged as an unrivaled approach to observe mechanochemical reaction mechanisms. Current approaches to *in situ* XRPD monitoring of ball-milling transformations all rely on synchrotron radiation, which is a method with limited accessibility. Moreover, reaction monitoring by XRPD is largely limited to crystalline materials, and analysing systems with large quantities of unknown or amorphous phases is a challenge. In contrast, reaction monitoring by *in situ* Raman spectroscopy is a more accessible bench-top technique, capable of providing real-time information on supramolecular and covalent changes during a mechanochemical reaction. Unlike XRPD data, however, the structure-based interpretation of Raman spectra of multicomponent crystalline materials is not straightforward. As mechanochemistry becomes increasingly popular and applied to more complex chemical transformations, development of more robust and accessible reaction monitoring approaches becomes of critical value.

**Fig. 1:**
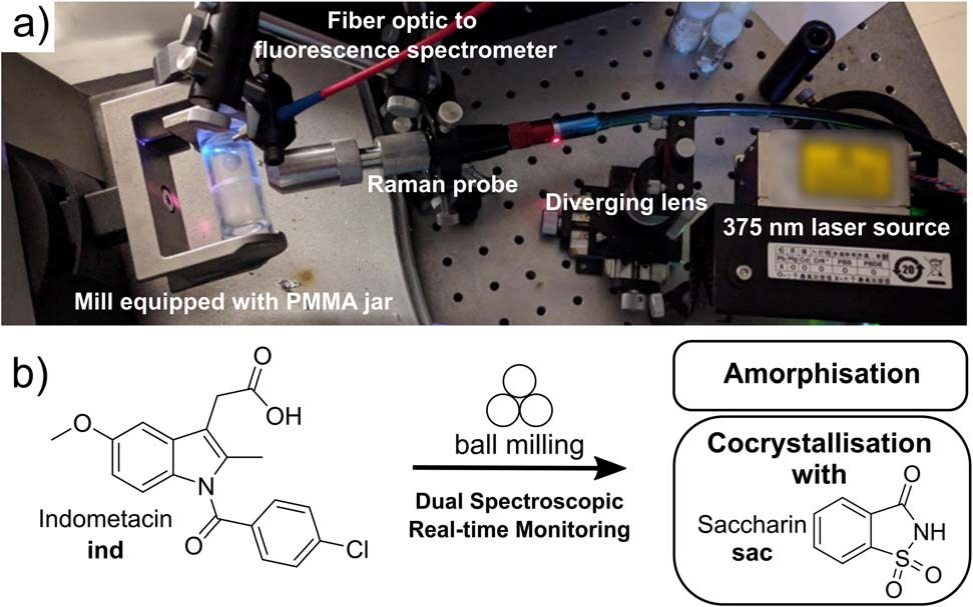
(a) Instrumental setup for tandem time-resolved *in situ* monitoring of milling reactions using solid-state fluorescence emission and Raman spectroscopies. (b) Model mechanochemical transformations monitored in this work: amorphisation and cocrystallisation of indometacin (ind).

Here, we demonstrate an integrated dual-spectroscopy approach (Fig. 1a) for time resolved *in situ* monitoring of milling reactions using readily accessible bench-top Raman and visible fluorescence emission spectrometers. The combination of these spectroscopic techniques with computational methods provided an opportunity to explore the potential and limitations of using highly accessible spectroscopy and modelling approaches to understand mechanochemical reaction mechanisms. Specifically, periodic time-dependent density-functional theory (TD-DFT) calculations enabled the association of spectroscopic data with the underlying molecular and extended solid-state structures of reactants and products.

*In situ* spectroscopy provides an attractive opportunity to detect changes in molecular structures and their solid-state environments regardless of crystallinity – including the direct observation of amorphous materials. We integrated the fluorescence spectroscopy instrumentation into an established Raman spectroscopy monitoring setup,^6^ enabling tandem time-resolved *in situ* monitoring of milling reactions without the need for synchrotron radiation. Both Raman and fluorescence spectroscopies are non-destructive, provide simultaneous sensitivity to changes in molecular and extended solid-state structure of crystalline and amorphous solids, and can be conducted with highly accessible, low-cost benchtop experimental setups capable of high sensitivity and acquisition speeds.^7–10^

This dual spectroscopy method is introduced as a means to investigate transformations of organic solids, specifically polymorphic transitions, cocrystallisation, as well as for the direct, real-time observation of amorphous material during milling.^11,12^ Amorphisation is a particularly important problem in mechanochemical processing of active pharmaceutical ingredients (APIs). Direct and real-time observation of amorphous phases during milling has remained elusive due to the well-known difficulty of quantifying amorphous content in real time by XRPD without an internal, impact-resistant diffraction standard.^13–16^ Research into the detection of amorphous content in milling reactions has so far largely focused on Rietveld refinement or atomic pair distribution function analysis of synchrotron X-ray data.^17,18^ Here, we apply both real-time Raman and fluorescence spectroscopies to directly follow amorphous phases during milling in the laboratory.

Indometacin (ind, Fig. 1b) was chosen as a target, as it is pharmaceutically relevant, and provides a rich landscape of solid forms that exhibit solid-state fluorescence and undergo diverse mechanochemical transformations, including amorphisation, polymorph transformations, and cocrystallisation.^19–21^ Separate fluorescence^22^ or Raman spectroscopy approaches have been reported for distinguishing and quantifying ind solid forms in static systems, including the amorphous form (am-ind); the thermodynamically stable γ-polymorph (γ-ind); the metastable α-polymorph (α-ind), and the saccharin (sac) cocrystal (ind-sac).^23–32^

Despite recent interest in mechanically-induced changes in luminescence *via* aggregation or amorphisation,^33–37^ the use of real-time luminescence for monitoring ball milling reactions has remained largely unexplored and limited to fluorescence coincidentally observed in Raman spectra.^38^ This is most likely because fluorescence spectroscopy data is challenging to interpret, owing to the broad emission profiles of organic molecules and difficulty in relating spectroscopic responses to solid-state and molecular structures. A possible opportunity to overcome this limitation, and open spectroscopic monitoring of solids to direct and *ab initio* structural interpretation, is offered by periodic density-functional theory (DFT) approaches. In principle, periodic DFT calculations should facilitate the development of an integrated understanding of molecular and supramolecular transformations taking place during a mechanochemical reaction, by enabling the interpretation of spectroscopic (*e.g.*, fluorescence, Raman, infrared, solid-state nuclear magnetic resonance – ssNMR) and XRPD monitoring outputs.

As a step towards developing such integrated understanding, we now evaluate the ability of periodic time-dependent DFT to interpret *in situ* fluorescence and Raman spectroscopy data, using calculations of ssNMR spectra as a validation benchmark. Specifically, periodic DFT was used to assign observed Raman data to vibrational motions within the crystal structure, and our recently developed method for calculating solid-state fluorescence emission spectra of crystalline materials (using the periodic implementation of TD-DFT in the CASTEP program)^39,40^ allowed us to effectively reproduce experimentally measured fluorescence spectra. At the same time, it is shown that the observed enhancement and wavelength shift of emission of ind in the solid state, compared to solution, is a result of conformational constraints imposed by the crystalline periodic environment. The herein reported computational analysis of vibrational spectra is, to the best of our knowledge, the first attempt to achieve structural insight into solid phases participating in mechanochemical reactions through real-time spectroscopy.

## Results and discussion

### Design of the tandem spectroscopic reaction monitoring setup

Indomethacin exhibits strong fluorescence in solid forms, but is poorly emissive in solution.^41^ The identities of solid forms γ-ind, sac, and ind-sac used in this work were verified by XRPD (Fig. S1–S3†), while excitation and emission spectra of these solids suggest an optimal excitation wavelength (*λ*) near our 375 nm laser wavelength (see ESI, Fig. S5†), with sac exhibiting a very weak emission and γ-ind displaying a considerably stronger one. The emission maximum of γ-ind is near 472 nm, while ind-sac is slightly red-shifted to *ca.* 498 nm, with a higher emission intensity (see ESI, Fig. S5†). The respective emission lifetimes for γ-ind and ind-sac were measured as 1.8 ns and 5.6 ns (see ESI, Table S1†). Bandgaps were determined from ultraviolet-visible (UV-Vis) spectroscopy data (see ESI, Fig. S6–S11†) and are statistically equivalent for both γ-ind and ind-sac (see ESI, Table S2†). The similarity in experimental emission wavelengths, bandgaps, and emission lifetimes suggests similar mechanism of optical excitation and emission found in γ-ind and ind-sac. The lack of fluorescence emission above 700 nm suggested the use of a 785 nm excitation laser for Raman studies, enabling an effective tandem spectroscopic approach. A fluorescence excitation source consisting of a 375 nm laser with a diverging lens to spread the excitation light, and fiber-optically-coupled spectrometer were integrated into our existing in-house real-time Raman spectroscopy setup for monitoring ball-milling reactions using optically transparent poly(methylmethacrylate) (PMMA) milling jars (Fig. 1b).^6^

The Raman spectra of γ-ind, α-ind, am-ind, sac, and ind-sac were consistent with previous reports^25,26,28^ and suggested that focusing on the 1500–1800 cm^−1^ region should enable reaction monitoring with minimal interference from the PMMA milling jar (Fig. S12 and S13†).

### *In situ* monitoring of the cocrystallisation of indometacin and saccharin

**Fig. 2:**
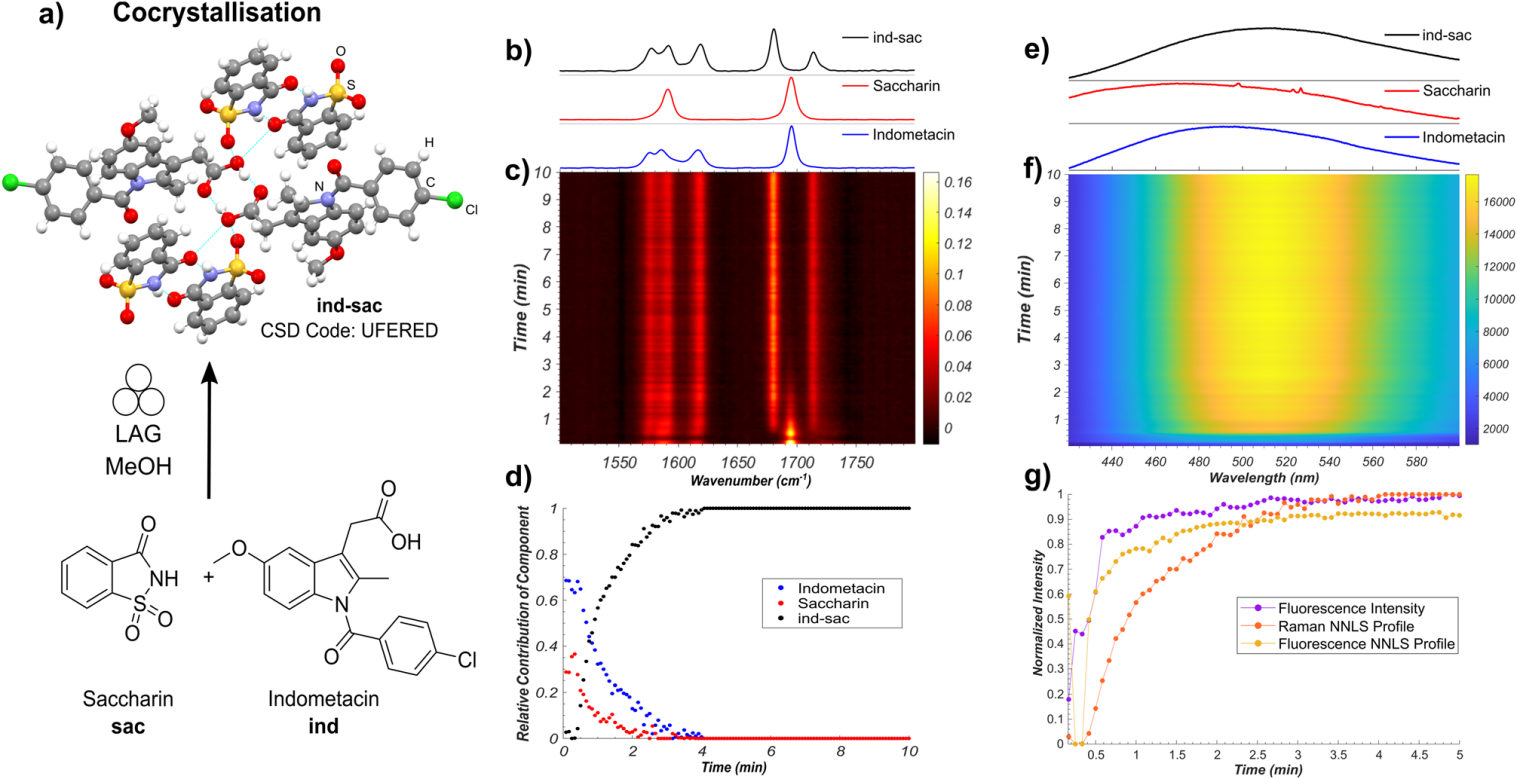
(a) Illustration of the monitored mechanochemical cocrystallisation of solid ind and sac upon LAG in the presence of MeOH. The atoms of the ind-sac crystal structure are shown in: red: oxygen, blue: nitrogen, green: chlorine, grey: carbon, white: hydrogen, and yellow: sulfur. Hydrogen bonds are displayed with pale blue lines. (b) Normalised Raman spectra of ind, sac, and ind-sac. (c) Time-resolved Raman spectra acquired during the mechanochemical synthesis of ind-sac. (d) Relative amounts of ind, sac, and ind-sac estimated using non-negative least squares fitting of the *in situ* Raman dataset. (e) Normalised fluorescence emission of ind, sac, and ind-sac. (f) Time-resolved fluorescence emission spectra acquired during the mechanochemical synthesis of ind-sac. (g) Comparison of the estimated formation of ind-sac*via* NNLS fitting of both Raman and fluorescence data sets and normalised maximum fluorescence intensity.

Our first target was the cocrystallisation of ind and sac, previously reported to proceed rapidly by liquid-assisted grinding (LAG)^24^ in the presence of methanol (MeOH) (Fig. 2a). Fluorescence spectroscopy monitoring of the milling process revealed a rapid increase in both emission intensity and a red shift of the emission maximum by approximately 30 nm to ∼498 nm, consistent with the formation of ind-sac.^22^ While the presence of MeOH could lead to the appearance of a known solvate of ind (CSD code BANMUZ),^42^ the fluorescence emission data indicated that the conversion to the cocrystal proceeds without any other solid phases, and was quantitative within 5 minutes.

To verify the reaction profile indicated by fluorescence emission spectroscopy measurements, we compared the time-dependent normalised luminescence intensity at the fluorescence emission maximum with the reaction profiles determined by non-negative least squares (NNLS) fitting of both *in situ* Raman spectroscopy (Fig. 2b–d and S14†) and fluorescence emission (Fig. 2e–g and S15†).^6^ The resulting agreement (Fig. 2b–g) is reasonable, indicating that simple monitoring of luminescence intensity can be used to directly estimate the time-dependent reaction profile over time. This provides a simple new route to spectroscopically follow mechanochemical reactions, using a readily accessible experimental setup. Moreover, the high fluorescence intensity enables spectra acquisition rates that are considerably faster (10–500 ms) compared to either XRPD or Raman spectroscopy. Fluorescence spectroscopy is less prone to stochastic changes in intensity as the experiment measures a larger area of the sample, making it less affected by motions of the milling assembly.^43^ However, compared to Raman spectroscopy, both fluorescence intensity measurement and the NNLS fitting profile suggest a slightly faster conversion to the ind-sac cocrystal. We hypothesise that the strong absorption of light at the excitation wavelength by the sample results in a shallow penetration depth into the solid, with fluorescence emission therefore primarily occurring at particle surfaces. As reactions between solid particles are expected to occur at the surface,^44^ the apparent reaction kinetics obtained from fluorescence would be faster than that obtained from Raman spectroscopy, a method with greater sample penetration depth. Accordingly, it may be difficult to detect low fractions of ind in inhomogeneous solids using fluorescence and factors such as particle size could significantly affect the emission.^22^ The similarity of fluorescence emission profiles between ind and ind-sac exacerbates the challenge of modeling *in situ* spectra as the sum of the spectra of pure components using the NNLS fitting method, as evidenced by patterns in the calculated residuals (see ESI, Fig. S15†).

Nevertheless, fluorescence emission spectroscopy provided an accessible, simple means to monitor the kinetics of mechanochemical cocrystallisation, due to significant shifts in emission wavelength and intensity throughout the process. The completeness of conversion was verified by XRPD analysis of the product immediately after milling, revealing Bragg reflections consistent with the reported ind-sac structure (CSD code UFERED),^24^ and no starting materials (see ESI, Fig. S1 and S3†). Full conversion was also confirmed by comparing XRPD (see ESI, Fig. S3†), ^13^C cross-polarisation magic angle spinning (CP-MAS) ssNMR data (see ESI, Fig. S16†), and Fourier-transform infrared spectroscopy (FT-IR) spectroscopy (see ESI, Fig. S17†) for the milling product to those for a solution-made sample of ind-sac (see ESI, Fig. S19†).^45^ The formation of ind-sac by LAG without observable participation of bulk amorphous or solvate phases was confirmed by separate real-time synchrotron XRPD monitoring at the Deutsches Elektronen-Synchrotron (DESY) PETRA III P02.1 beamline (see ESI, Fig. S18†).

### Spectra interpretation using periodic DFT: Raman, FT-IR, and NMR spectroscopies

To link the real-time spectroscopic measurements to structures of participating solid phases, we have used CASTEP16 ^40^ plane-wave DFT calculations to simulate the Raman, infrared, and ssNMR spectra from crystal structures of γ-ind, sac and ind-sac solids. Periodic DFT simulations of both FT-IR and Raman spectra were in reasonable agreement with the experiment for γ-ind, sac, and ind-sac (see ESI, Fig. S19–S24†) enabling the qualitative assignment of most Raman peaks to specific normal modes. While periodic DFT is required to incorporate the effects of supramolecular interactions in solid-state structures, less computationally intense and more accessible gas-phase DFT calculations in Gaussian 16 ^46^ can help identify which functional groups are responsible for vibrational modes which shift over the course of a reaction. The assignment of specific Raman bands to vibrational modes was performed for both ind and sac using periodic DFT, resulting in superior correlation to experimental spectra relative to gas phase calculations (see ESI, Fig. S25 and S26†). These calculations facilitated the assignment of Raman active vibrations above 1650 cm^−1^ as due to carbonyl stretching, and those between 1500 and 1650 cm^−1^ as C–C stretching of the aromatic rings in ind and sac (see ESI, Fig. S27 and S28†). In case of pure solids, the carbonyl stretches of both γ-ind and sac overlap near 1696 cm^−1^, but are observed to diverge in the ind-sac cocrystal. The application of periodic DFT enables the assignment of the absorption bands which shifted to higher wavenumbers (1714 cm^−1^) as corresponding to the carbonyl stretching vibrations of sac, while the ind carbonyl stretching was found to shift to lower wavenumbers (1681 cm^−1^) (see ESI, Fig. S29†). The periodic DFT calculations are in reasonable agreement with the experimental solid-state Raman and FT-IR data for the region of 1500 to 1800 cm^−1^, with the calculated vibrational frequencies falling within 20 cm^−1^ for the C–C ring stretching vibrations and within 45 cm^−1^ for the carbonyl stretching without the use of a scaling factor. The magnitude of these differences is consistent with those reported in the literature, without the use of empirical scaling factors.^47^ The periodic DFT correctly predicted the changes in vibrational frequencies between the different crystal structures with remarkable agreement (<5 cm^−1^) to experimental results. During the reaction, the carbonyl stretch of ind decreases by 15 cm^−1^, from 1696 cm^−1^ to 1681 cm^−1^, while a shift of 17 cm^−1^ is predicted from periodic DFT. The carbonyl stretch of sac shifts from 1696 cm^−1^ to 1714 cm^−1^ an increase of 18 cm^−1^ with DFT predicting an increase of 19 cm^−1^. The bands corresponding to the C–C ring stretching vibrations were predicted not to shift significantly (<3 cm^−1^), which is consistent with the experimental spectra. Similar trends can be observed in the FT-IR data which suggests that periodic DFT is a very powerful tool for understanding changes in vibrational frequencies which occur during reactions (Fig. S19–S21†).

Finally, our periodic DFT modelling approach was validated by comparing the experimental ^13^C CP-MAS ssNMR chemical shifts for all three phases, with the chemical shifts calculated using the GIPAW method implemented in CASTEP.^48^ Reasonable agreement between the calculated and experimental values (<5 ppm difference) (Table S3, Fig. S30–S32†) for all but three ^13^C signals signified the accuracy of our computational model for the crystal forms of ind. A comparison of simulated ^13^C chemical shifts for ind-sac and either sac or γ-ind revealed that the most significant differences (see ESI, Table S4†) were consistent with the carbon atoms adjacent to short contact interactions in the cocrystal structure (see ESI, Fig. S33†). This observation emphasises the accuracy of the GIPAW method in quantifying the effect of supramolecular interactions on the ssNMR spectra of molecular crystals.

### Using TD-DFT to understand the solid-state fluorescence of indometacin

Beyond allowing the association of experimental data to structures of participating crystalline phases, periodic TD-DFT calculations should also enable a deeper understanding of the mechanism underlying fluorescence behavior of solid-state ind. Switching between solid γ-ind and the ind-sac cocrystal was reported^22^ to have a significant effect on emission properties of ind, a molecule with very low fluorescence quantum yield in solution,^41^ representing an example of emission enhancement by crystal lattice effects. Previous work has shown that using cocrystallisation can alter optical and emission properties of organic chromophores through different mechanisms, notably forming or breaking of π–π stacking interactions,^49^ or by direct orbital overlap between molecules.^50^

**Fig. 3:**
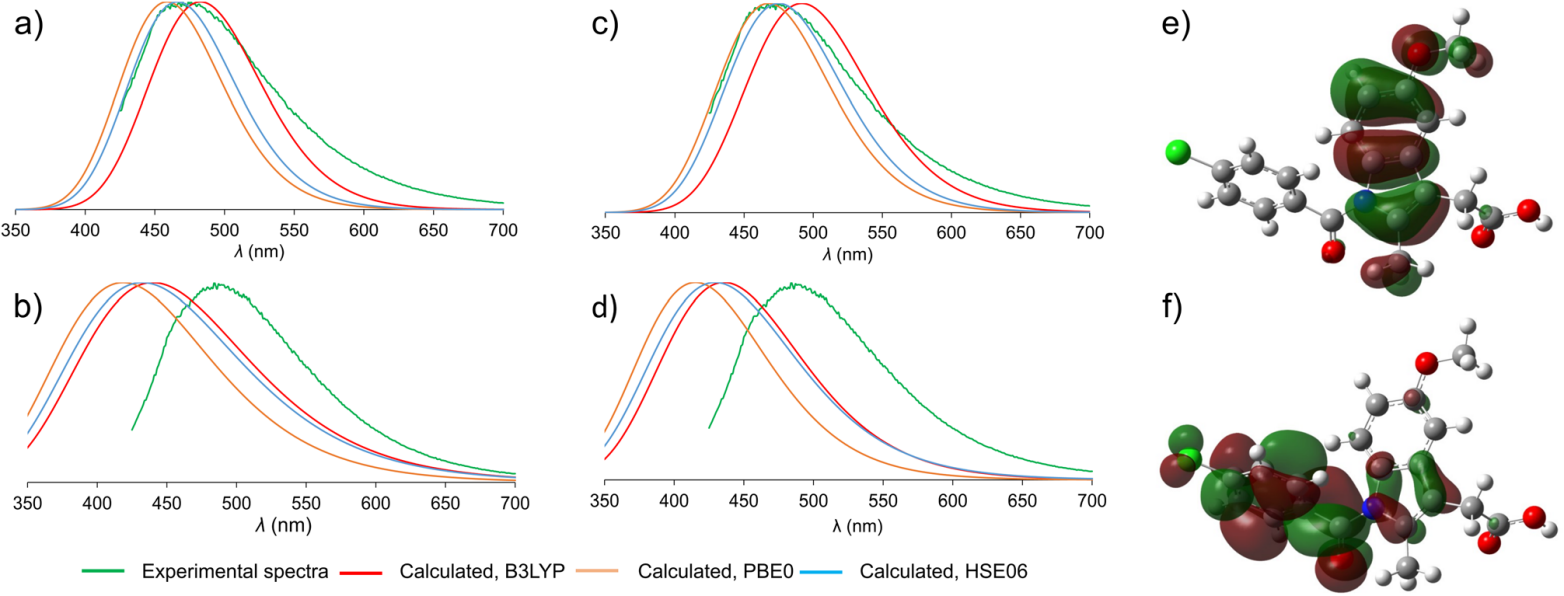
Comparison of the experimental (green) and simulated normalised emission spectra. The experimental spectra are shown in green, simulated spectra are colored depending on the hybrid functional used for the single point TD-DFT calculation: red – B3LYP; orange – PBE0; blue – HSE06. (a) Emission spectra of γ-ind, TD-DFT optimisation with dispersion-corrected PBE; (b) emission spectra of ind-sac, TD-DFT optimisation with dispersion-corrected PBE; (c) emission spectra of γ-ind, TD-DFT optimisation with LDA; (d) emission spectra of ind-sac, TD-DFT optimisation with LDA; (e) HOMO orbital of an ind molecule, showing electron density localised on the indole fragment; (f) LUMO orbital of an ind molecule, showing most electron density shifted towards the benzoyl moiety.

To understand the emission properties of ind, we turned to TD-DFT simulations of solid γ-ind and ind-sac. While fluorescence emission of individual molecules can be readily simulated by molecular TD-DFT, cubic scaling of the calculation with system size quickly makes such approach prohibitive for modelling solid-state emission by cluster expansion. As an alternative, we developed a method for simulating solid-state fluorescence emission spectra of crystalline materials using the periodic implementation of TD-DFT in CASTEP.^39^ Since our method explicitly operates in a plane-wave basis set, it is capable of studying the role of non-covalent interactions, orbital overlap, and conformational effects on the emission of molecular crystals. The simulated emission spectrum of γ-ind was in excellent agreement with experiment, demonstrating the power of periodic DFT in modelling the emission behavior of crystalline solids (Fig. 3). Notably, our calculations showed consistency with respect to the choice of DFT functionals: we have tested two methods for geometry optimisation of electronic excited state (LDA and dispersion-corrected PBE)^51–53^ combined with each of the three hybrid functionals (B3LYP,^54–57^ HSE06 (ref. 52 and 58) and PBE0 (ref. 59 and 60)) for single point calculation of excitation energies. All six combinations of functionals resulted in emission maxima within 0.2 eV of each other, which corresponds to 22 nm variation in *λ*_max_. In terms of orbital contribution, it was found that the emission process originates from the S_1_ excited state, which is dominated by the LUMO → HOMO electron transition.

Next, we turned our attention to simulating the emission spectrum of the ind-sac cocrystal. The simulated emission spectra calculated with different functionals were, once again, in close agreement with each other (Fig. 3b and d). In terms of agreement with the experimental emission spectrum, the periodic TD-DFT calculations underestimated *λ*_max_ for ind-sac by 50–70 nm, which is less accurate than for γ-ind, but is reasonable given the complexity of modelling solid-state emission from a multi-component crystal. The electronic transformation responsible for the emission behavior of both γ-ind and the ind-sac cocrystal, according to periodic TD-DFT, was found to be HOMO and LUMO of ind, same as in the case of γ-ind. Consequently, orbitals of sac were not deemed responsible for the fluorescence emission of the cocrystal. Fully periodic calculations using a range-separated functional, are not accessible in CASTEP, so molecular TD-DFT calculations using the CAM-B3LYP functional were run in Gaussian 16. These calculations were performed on a cluster containing two ind and two sac molecules (see ESI, Fig. S34†). The electronic transition originating from a mix of HOMO−1(ind) and HOMO(ind) onto LUMO(ind) has a large oscillator strength of 0.2067, whereas the hypothetical charge transfer state from HOMO−1(ind) to LUMO(sac), has a negligible oscillator strength of just 0.0013. Neither periodic nor cluster calculations suggest a charge-transfer mechanism. However, the red-shift in fluorescence emission observed experimentally upon cocrystallisation of ind and sac was not reproduced by periodic DFT, emphasising the difficulty of modeling subtle differences in underlying electronic structure responsible for the change in fluorescence emission.

An important question to be answered in the context of ind fluorescence is the extremely weak emission in solution, contrasting the strong emission of solid γ-ind. The strong solvent dependence of the Stokes shift of ind in solution was postulated to result from a dipolar singlet excited state which is produced by intramolecular charge transfer from the indole to the benzoyl group.^41,61^ The molecular TD-DFT calculations suggest that the electronic excitation of an isolated ind molecule is expected to be accompanied by a 40° rotation of the benzoyl group to an orientation perpendicular with respect to the plane of the indole system (see ESI, Fig. S35†). This can be explained by the redistribution of electron density from the indole system in the HOMO to the benzoyl group in the LUMO (Fig. 3e and f). For a molecule in an unconstrained environment, *e.g.* in solution, this rotation results in a significant reduction of the electronic transition dipole moment and quenching of the fluorescence emission.^62^ This is consistent with the established behavior of ind in solution.^41^ Conversely, in constrained environments of crystalline γ-ind and ind-sac, such a rotation is restricted to less than 10°, regardless of the functional used for TD-DFT geometry optimisation. Overall, our calculations show that the emissive behaviour of ind in the solid-state results from the close-packed crystal structure limiting the geometric distortions that a molecule can undergo in the electronic excited state.

### Milling amorphisation of indometacin

**Fig. 4:**
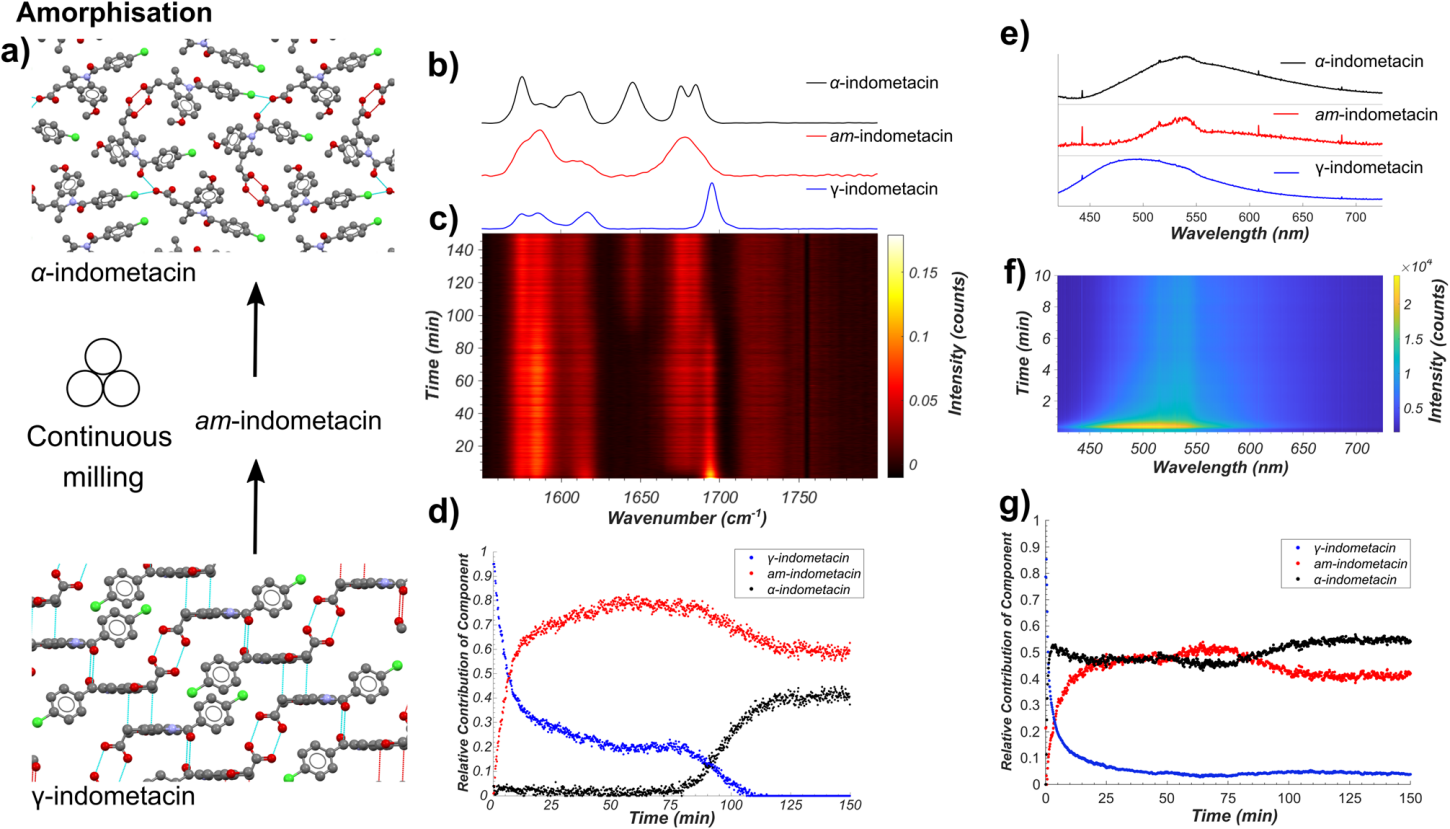
(a) Schematic of the monitored model transformation of neat milling solid γ-ind. The atoms of the indometacin crystal structures are displayed with the following colors: red: oxygen, blue: nitrogen, green: chlorine, grey: carbon, and white: hydrogen. (b) Normalised Raman spectra of γ-ind, α-ind, and am-ind synthesised *via* solution or melt protocols.^63,64^ (c) Time-resolved normalised Raman spectra acquired during the milling of γ-ind. (d) Relative amounts of γ-ind, α-ind, and am-ind estimated using non-negative least squares fitting of the *in situ* dataset using the reference spectra in (b). (e) Estimated fluorescence spectra of γ-ind, α-ind, and am-ind obtained from non-negative matrix factorisation of the real time fluorescence emission spectroscopy dataset. Due to the low emission intensity of the α- and am-forms the effect of detector baseline becomes significant. (f) Time-resolved fluorescence emission data acquired during the milling of solid ind, truncated to 10 minutes for clarity. For full data see ESI Fig. S36.† (g) Relative amounts of γ-, α-, and am-ind estimated using non-negative matrix factorisation of the *in situ* fluorescence emission dataset.

Next, we applied our tandem spectroscopic monitoring method to the amorphisation of ind by ball milling (Fig. 4a), previously explored *ex situ* by XRPD^63^ and Raman spectroscopy.^26,27^ Raman spectroscopy *in situ* monitoring of ball-milling commercial γ-ind (verified by XRPD, see Fig. S2†) using a shaker mill operating at 30 Hz reveals the participation of at least three distinct ind phases: γ- and α-ind, along with the amorphous form (am-ind) (Fig. 4b–d).^23,25–28^ Reference spectra for these solid ind phases were obtained experimentally, using a commercial sample of γ-ind, a freshly synthesised sample of α-ind precipitated from a solution in a mixture of ethanol and water mixture,^64^ and a sample of am-ind made by quenching of a melt of ind using liquid nitrogen.^65^ Analysis of real-time Raman spectroscopy data (Fig. 4b–d) was done following the previously reported approach,^6^ where each *in situ* measured spectrum is fitted by a NNLS procedure using a combination of Raman spectra of pure samples to provide an estimate of the relative composition of the reaction mixture. This revealed within 10 minutes the emergence of am-ind co-existing with γ-ind, followed by the appearance of α-ind after ∼75 min (Fig. 4b–d). These observations are consistent with a previous *ex situ* XRPD study, which suggested that milling produces a 1 : 1 mixture of α- and am-ind.^63^ Changes observed by real-time fluorescence monitoring of γ-ind amorphisation (Fig. 4e–g) can be explained by ind molecules being more able to adopt non-emissive conformations, resulting in a red shift and a reduction in intensity of emission, readily observed by NNLS fitting of *in situ* data. However, NNLS fails to adequately differentiate α- and am-ind which have similar fluorescence emission profiles (see ESI, Fig. S36†). Monitoring the maximum emission intensity of each spectrum, however, does reveal a slight increase in emission intensity corresponding to the formation of α-ind, which is known to be slightly more emissive than am-ind (see ESI, Fig. S37†).^22^ The projected gradient method^66^ for non-negative matrix factorisation (NMF)^67^ was applied to simultaneously estimate both the component spectra and their associated profiles. The estimated component spectra are similar to both *ex situ* emission spectra (see ESI, Fig. S37†) and resemble the reaction profile estimated by Raman spectroscopy (see ESI, Fig. S38†). Accurately determining relative amounts of α- and am-ind only through fluorescence emission data remains challenging (Fig. 4f), highlighting the value of complementarity inherent to the dual monitoring approach. Unfortunately, the complexity of α-ind crystal structure, with three independent molecules in the unit cell and numerous intermolecular interactions, preclude the previously used cluster and periodic DFT methods both in terms of computational cost and interpretation of the result. Modeling the lack of crystalline order in am-ind is a fundamental challenge beyond the scope of this work.^68^ Both the Raman and fluorescence emission data sets are in agreement with a simple model proposed for amorphisation during mechanical alloying.^69^ In this model each impact of a milling ball can convert a small amount of γ-ind to am-ind, and the amount of material that has undergone impacts is expected to be an exponential in the form of e^−*kn*^, where *n* is the number of impacts and *k* is the amount of powder processed per impact. As milling is conducted at a constant frequency, the total number of impacts *n* is expected to be directly proportional to time (*t*), and the molar fraction (*α*) of γ-ind and am-ind can be expressed using the following equations:

1 *α*_γ-ind_(*t*) = e^−*kt*^

2 *α*_am-ind_(*t*) = 1 − e^−*kt*^

This model assumes a homogeneous mixture of solid phases. However, recent measurements on the milling amorphisation of trehalose suggest that amorphisation preferentially occurs at particle surfaces resulting in the formation of particles with crystalline cores and amorphous shells.^17^ The initial kinetics of the loss of γ-ind during amorphisation *via* milling are fairly consistent with an exponential decay function (eqn (1)) for both Raman spectroscopy and fluorescence emission data, although the rate constant obtained by fluorescence spectroscopy (*ca.* 0.52 s^−1^) is approximately double of that found from Raman spectroscopy (*ca.* 0.23 s^−1^) data (see ESI Table S5, Fig. S39 and S40†). We hypothesise that the fluorescence emission mostly originates from the amorphous shell on particle surfaces, while Raman spectroscopy has sufficient penetrating power to observe the slower conversion of crystalline cores. Such a hypothesis is consistent with the observed discrepancy in estimated fraction of γ-ind between the two techniques for 25–70 minutes of milling. This partially amorphised state of γ-ind cores with amorphous shells appears relatively kinetically stable until around 70 minutes of milling. At that point, crystalline α-ind begins to form, with a sigmoidal crystallisation profile similar to that expected from a kinetic model previously proposed for the conversion of β- to α-mannitol during milling.^18^ The nucleation of α-ind from am-ind above room temperature (30 °C) has previously been observed *ex situ*^70^ with these phases reported as being close in energy.^71^ In this case, however, it is unclear where in the particle or in which phase this transition preferentially occurs.

XRPD analysis of milled solid ind revealed an elevated baseline with broad signals of α-ind, consistent with the presence of amorphous material (see ESI, Fig. S2†). Attempt to characterise the sample by ^13^C CP-MAS ssNMR revealed signals identical to those for a separately synthesised sample of α-ind, but did not reveal any amorphous content (see ESI, Fig. S41†). The inability to observe am-ind by ssNMR suggests that the sample fully crystallised during the preparation of the ssNMR measurement a conclusion also supported by FT-IR measurements (see ESI, Fig. S42†). The recrystallisation of amorphous samples from ball milling has been previously been observed by DSC and is hypothesized to occur due to nano-sized crystalline cores in the sample.^17^ This highlights the need for real time approaches to understand the behaviour of amorphous solids during mechanochemical processes and for the synthesis of amorphous formulations of APIs.^72^

## Conclusions

In summary, we have demonstrated a novel bench-top tandem spectroscopy approach to follow mechanochemical ball-milling processes, based on simultaneous combination of Raman and fluorescence emission spectroscopies. By using the active pharmaceutical ingredient indomethacin as a model system, we demonstrate the utility of this tandem spectroscopy approach for monitoring transformations between crystalline and amorphous phases, as well as cocrystallisation by milling. Solid-state fluorescence emission spectroscopy is capable of real-time monitoring of milling reactions and provides information which is complementary to and consistent with Raman spectroscopy. The herein presented cost-effective tandem benchtop *in situ* monitoring technique offers an opportunity to follow and understand mechanochemical reaction mechanisms by revealing reaction kinetics and enabling the observation of amorphous phases where rapid relaxation prohibits the use of *ex situ* techniques. Interpretation of spectroscopic data using periodic DFT and TD-DFT calculations is at an early stage of development, but already provides a means to link real-time spectroscopic measurements to structural changes in the solid state, and to facilitate the use of spectroscopy to identify changes in supramolecular interactions. Whereas the presented dual spectroscopic methodology is readily applicable for studies of mechanochemical reactivity, the ability to use theoretical approaches for full structure-based interpretation of spectroscopic data remains limited to systems in which crystal structures of participating substances are known. It is likely, however, that the future development of advanced theoretical techniques, such as crystal structure prediction (CSP)^78^ and structural modelling of amorphous materials,^79^ will enable such comprehensive interpretation even for materials whose structures are yet to be determined. Overall, by stepping beyond spectroscopic fingerprinting, the herein presented work opens the door to spectroscopy-only techniques that can provide interpretable insights without requiring structural information from real-time synchrotron X-ray diffraction.

## Methods

All chemicals were purchased from Sigma-Aldrich and used without further purification, including solid indometacin which was found to be in the γ-form. Milling experiments were conducted using a RETSCH MM400 operating at 30 Hz, with a 15 mL volume PMMA milling jar and a single 3 g zirconia ball.

### Ball milling synthesis

The cocrystal ind-sac was prepared by milling 198 mg (0.55 mmol) γ-ind with 101 mg (0.55 mmol) of sac in the presence of 20 μL of MeOH. Neat milling of γ-ind to produce am-ind and α-ind was conducted using 198 mg (0.55 mmol) γ-ind.

### Solution- and melt-based syntheses

Amorphous ind was synthesised according to a literature procedure,^65^ by heating 300 mg of γ-ind above 165 °C and pouring the resulting melt into liquid nitrogen, forming an amorphous mass which was ground into a powder using a mortar and pestle.

Samples of α-ind were synthesised using a modified literature procedure,^64^ by dissolving 300 mg of γ-ind in 5 mL of ethanol at 80 °C before adding 10 mL of room temperature distilled water and filtering the resulting precipitate.

### Fluorescence, FT-IR, XRPD, UV-Vis, lifetimes, and ss-NMR characterisation

Preliminary fluorescence measurements were performed in clear polystyrene 96-well microplates, with sample densely packed into each well, and loaded into a Biotek Synergy 2 multi-mode microplate reader. All fluorescence lifetime measurements were conducted on a Horiba DynaMyc fluorescence lifetime mapping microscope equipped with a DeltaDiode-375L light source. Fluorescence lifetime data was fitted using a single exponential function. Fourier-transform infrared attenuated total reflectance (FT-IR-ATR) were measured on a Bruker Vertex 70 spectrometer with a RockSolid interferometer from 3500 cm^−1^ to 400 cm^−1^. X-ray powder diffraction (XRPD) patterns were collected using a Bruker D2 Phaser powder diffractometer equipped with a CuK_α_ (*λ* = 1.5418 Å) source, nickel filter and Lynxeye detector. Ultraviolet-visible (UV-Vis) measurements were performed on a Lambda 750 UV/Vis/NIR spectrometer from PerkinElmer. BaSO_4_ (ACS) was used as a standard for instrumental calibration (autozero correction). Samples were filled into a 1 cm^3^ quartz cuvette. Full spectra were recorded in reflectance in the range of 2500–300 nm with 5 nm intervals and between 620 and 300 nm with 0.5 nm intervals for calculating the band gap. ^13^C solid-state NMR spectra were collected using a Varian VNMRS 400 MHz NMR spectrometer, with a magic angle spinning rate of 14 kHz using a tancpx pulse sequence and calibrated with respect to the carbonyl signal of α-glycine signal at 176.4 ppm.

### Real-time synchrotron diffraction measurements

*In situ* X-ray diffraction measurements were collected at the Deutsches Elektronen-Synchrotron (DESY) PETRA III P02.1 beamline at an X-ray wavelength of 60 keV (*λ* ∼ 0.207 Å) with a 1 × 1 mm^2^ collimated X-ray beam and a PerkinElmer 2D area detector operating at a time resolution of 10 seconds and a modified RETSCH MM400. All 2D XRPD patterns were integrated using Dioptas.^73^ Sequential Rietveld analysis was performed in TOPAS-Academic V5.^74^ The instrumental peak profile was determined using a silicon standard measured under identical experimental conditions.^75^ For visualisation purposes, datasets were baseline corrected, truncated, and plotted using custom scripts in MATLAB R2018a with PMMA baseline subtraction performed using the Sonneveld and Visser algorithm.^76^

### *In situ* Raman spectroscopy

All Raman spectra were collected by a RamanRxn1™ analyser by Kaiser Optical Systems Inc. every 5 seconds using a 785 nm laser. Spectra were dark-subtracted and intensity corrected using the Holograms® software package before being processed. Pure samples of starting materials and products were loaded on glass slides and measured. *In situ* datasets were subsequently imported into MATLAB2020a and baseline corrected using the Sonneveld and Visser algorithm,^76^ truncated to the limits shown in the data sets, and normalised using vector normalisation (L2 norm). Background subtraction of the PMMA milling jar was performed by performing a linear regression of a previously recorded PMMA spectrum to each *in situ* spectrum and subtracting the PMMA spectrum. After these corrections, the data was analyzed *via* NNLS, where *in situ* collected spectra were fitted as a sum of the normalised component spectra using a non-negative linear least squares algorithm (“lsqnonneg” in MATLAB) and profile estimates were normalised by setting the sum of all components in each spectrum to one.^6^ Kinetic analysis was performed using the Curve Fitting Tool in MATLAB 2020a using the equations given in the ESI.†

### Real-time fluorescence emission spectroscopy

Fluorescence measurements were conducted using a Coherent OBIS 375 nm LX 50 mW excitation source and fiber-optically coupled QE65000 spectrometer from Ocean Optics. Pure samples of starting materials and products were loaded on glass slides and measured. *In situ* datasets were subsequently truncated to the limits shown in their respective figures and plotted using custom scripts in MATLAB R2020a. NNLS profiles were obtained in an identical manner as described for Raman spectra. Normalised fluorescence intensity values were calculated by subtracting the minimum value of each *in situ* spectrum and dividing the spectrum by the maximum intensity value.

### Periodic density-functional theory calculations of fluorescence emission spectra

All periodic DFT calculations were performed in CASTEP 16.11. Calculation of solid-state fluorescence spectra of ind and ind-sac was performed using our previously described procedure.^39^ The experimental crystal structures were converted to CASTEP input format using the program cif2cell.^77^ Initially the crystal structures were then geometry optimised in their ground state electronic configurations using either LDA functional or PBE functional combined with Grimme D2 dispersion correction. The plane-wave basis set was truncated at 750 eV cut-off combined with norm-conserving pseudopotentials, while the 1^st^ electronic Brillouin zone was sampled with 2π × 0.03 Å^−1^*k*-point spacing. The crystal structures were geometry-optimised with respect to unit cell parameters and atom positions, subject to the space group symmetry constraints. Convergence was determined using the following criteria: maximum energy change: 10^−5^ eV per atom; maximum atomic force: 0.05 eV Å^−1^; maximum atomic displacement: 10^−3^ Å, maximum residual stress: 0.05 GPa. The optimised unit cell parameters were kept fixed through all the subsequent steps of the fluorescence calculation.

CASTEP TD-DFT calculations can only be performed at one *k*-point in the Brillouin zone. The *k*-point offering the best approximation to the converged *k*-point grid was selected by calculating the singlet–triplet energy difference for a series of *k*-points. The special *k*-point found to accurately reproduce the singlet–triplet energy difference for the converged *k*-point grid, analogous to the idea of the so-called Baldereschi point^60^ was found at (1/4; −3/8; 1/8) for γ-ind and at (1/4; 1/8; 1/8) for ind-sac. Next, excited state TD-DFT calculations were performed. In the case of γ-ind the 1^st^ excited state was optimised, which corresponded to the HOMO–LUMO transition on indometacin. In the case of ind-sac, the 1^st^ TD-DFT excited state involved transition from HOMO(ind) to LUMO(sac), which corresponded to a low-intensity charge transfer (CT) state, known as an artefact of TD-DFT. With the aid of molecular range-separated TD-DFT calculations (see below), this was ruled out as an incorrect solution, and instead a higher rank TD-DFT excited state corresponding to the HOMO(ind)–LUMO(ind) transition was chosen. That way both γ-ind and ind-sac follow the same mechanism of fluorescence emission.

The selected excited states were geometry-optimised using CASTEP TD-DFT module. Same input settings and convergence criteria were used here as for ground-state geometry-optimisation, except for unit cell parameters which were kept fixed. The final step of the fluorescence calculation was a single point TD-DFT calculation using each of the three functionals: PBE0, B3LYP and HSE06. The hybrid calculations were performed both on the ground state- and TD-DFT-optimised geometries, the energy difference between these two geometries being used to approximate the width of the spectral line, approximated by the Gaussian curve.

### Periodic DFT calculations of vibrational and NMR spectra

The ground-state optimised structures for the fluorescence calculations were used as a starting point for the Raman and NMR calculations.

For the Raman calculation the crystal structures were reoptimised with a tighter atomic force convergence criterion of 0.01 eV Å^−1^. Further, the standard and fine FFT grid scales were changed from their default values to 2 and 3, respectively. The vibrational frequencies at the *Γ* phonon *q*-point were calculated using the density-functional perturbation theory (DFPT) approach. The polarisability tensors were then calculated for the Raman-active modes. Spectra were simulated as using Gaussian functions for each Raman active vibration, using the calculated Raman frequencies, scattering activities, and a peak width of 6 cm^−1^. All spectra were normalised to *via* the highest intensity for plotting.

FT-IR spectra were simulated as a summation of Gaussian functions for IR active vibrational modes using a peak width of 15 cm^−1^, the calculated vibrational frequency, and relative peak amplitudes obtained from the CASTEP calculation. All spectra were normalised to a maximum intensity of 0.5 and converted into transmittance for comparison to experimental spectra.

The NMR parameters were calculated using the gauge including projector augmented waves (GIPAW) method. The plane-wave basis set cut-off was increased to 1000 eV, the standard and fine FFT grid scales were set to 2 and 3, respectively, and ultrasoft on-the-fly generated pseudopotentials were used.

### Molecular DFT calculations

Vibrational spectra were simulated using gas phase DFT calculations run using Gaussian 16W using the PBE and B3LYP functionals and the 6-311G(d,p) basis set using “tight” optimisation convergence criteria. Gas phase spectra calculation and vibrational modes visualisation were conducted in GaussView 6.1 using the default settings. Molecular TD-DFT calculations for an isolated ind molecule were performed at the CAM-B3LYP/6-311G(d,p) level of theory. The 1st electronic excited state was geometry optimised with the default convergence criteria, and then a 360° torsion angle scan in 10° steps was performed to describe the rotation of the benzoyl part of the molecule with respect to the indole fragment. The oscillator strength for the electronic transition between the 1st excited and the ground state was computed at each step of the torsion angle scan.

## Data availability

Data supporting this article has been provided as ESI.†

## Author contributions

Experiments were conducted and processed by P. A. J., L. S. G. with assistance from M. E., R. E. D., and T. F. Computations in the manuscript were performed by M. A. with assistance from A. J. M. The manuscript was written by P. A. J., M. A., and T. F. with contributions from all authors.

## Conflicts of interest

The authors declare no competing interests.

## Supplementary Material

SC-014-D3SC04082H-s001
