# Supplementary material for: Illuminating milling mechanochemistry by tandem real-time fluorescence emission and Raman spectroscopy monitoring
Source: Chem Sci. 2023 Oct 12;14(43):12121–32. doi: 10.1039/d3sc04082h (PMC10631231; doi:10.1039/d3sc04082h)
Supplement: SC-014-D3SC04082H-s001 [file SC-014-D3SC04082H-s001.pdf]

Electronic Supplementary Material (ESI) for Chemical Science.
This journal is © The Royal Society of Chemistry 2023

Electronic Supplementary Information for:

Illuminating mechanochemical reactions by tandem real-time fluorescence emission and Raman spectroscopic monitoring

Authors: Patrick A. Julien*, Mihails Arhangelskis*, Luzia S. Germann, Martin Etter, Robert E. Dinnebier, Andrew J. Morris* and Tomislav Friščić*

Table of Contents

1. X-ray Powder Diffraction 2
2. Experimental Setup 3
3. Fluorescence Excitation and Emission Spectra and Lifetime Measurements 4
4. Band-Gap Measurements 4
5. Raman Spectra of Starting Materials and Products 8
6. Fitting of *in situ* spectroscopy datasets for ball-milling cocrystallisation of ind and sac 9
7. Solid-State ^{13}C NMR and FTIR-ATR Spectroscopy 11
8. *In situ* Synchrotron Powder X-ray Diffraction Monitoring of Cocrystallisation by Milling 12
9. Comparison of Predicted and Experimental Vibrational Spectra 13
10. Comparison of Predicted and Experimental NMR Spectra 19
11. TD-DFT Orbital Transitions 23
12. Fitting of *in situ* spectroscopy datasets of indometacin amorphisation by milling 24
13. Kinetic modelling of amorphisation 26
14. Solid-State NMR and FTIR-ATR spectroscopy for indometacin amorphisation by ball-milling 29

1. X-ray Powder Diffraction

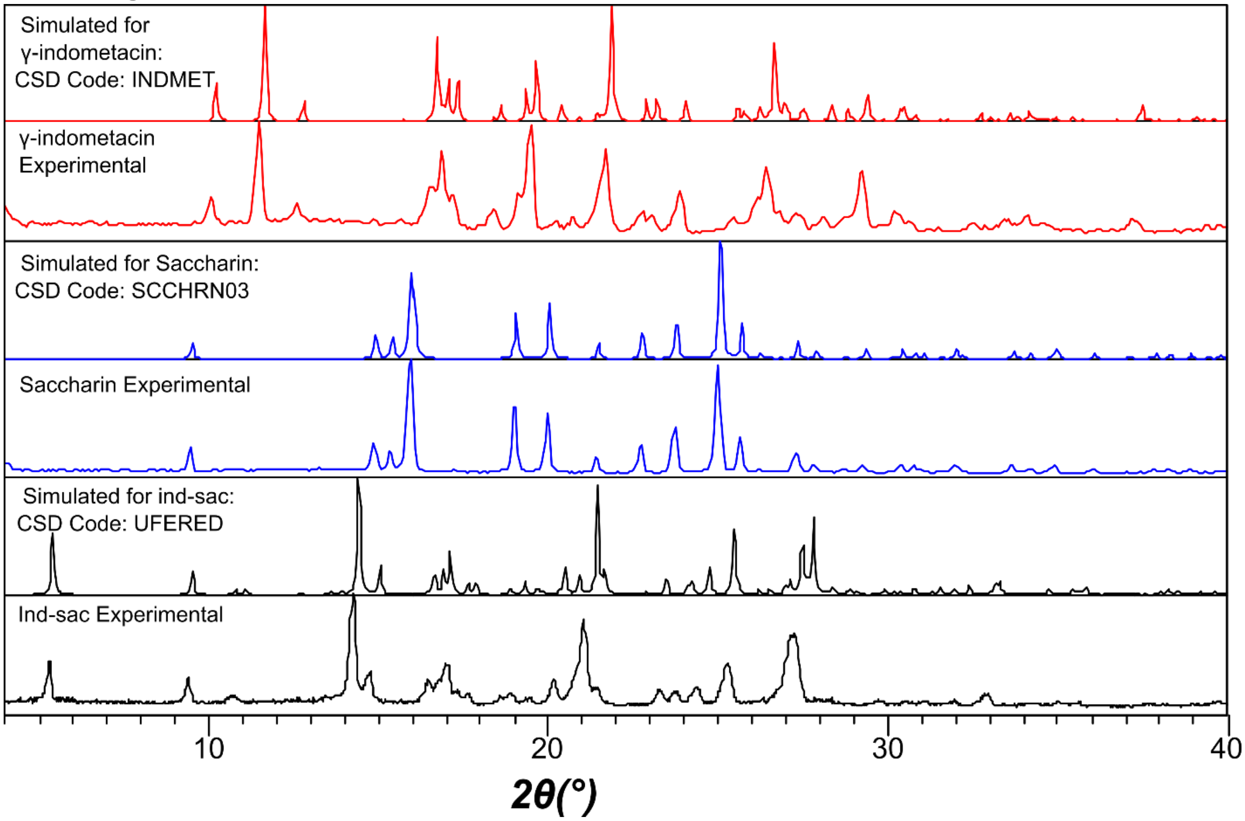

Figure S1. X-ray powder diffraction patterns of commercial **γ-ind** (red), **sac** (blue) and **ind-sac** (black) obtained from milling synthesis compared with the respective simulated XRPD patterns based on reported crystal structures (CSD codes INDMET and SCCHRN03).

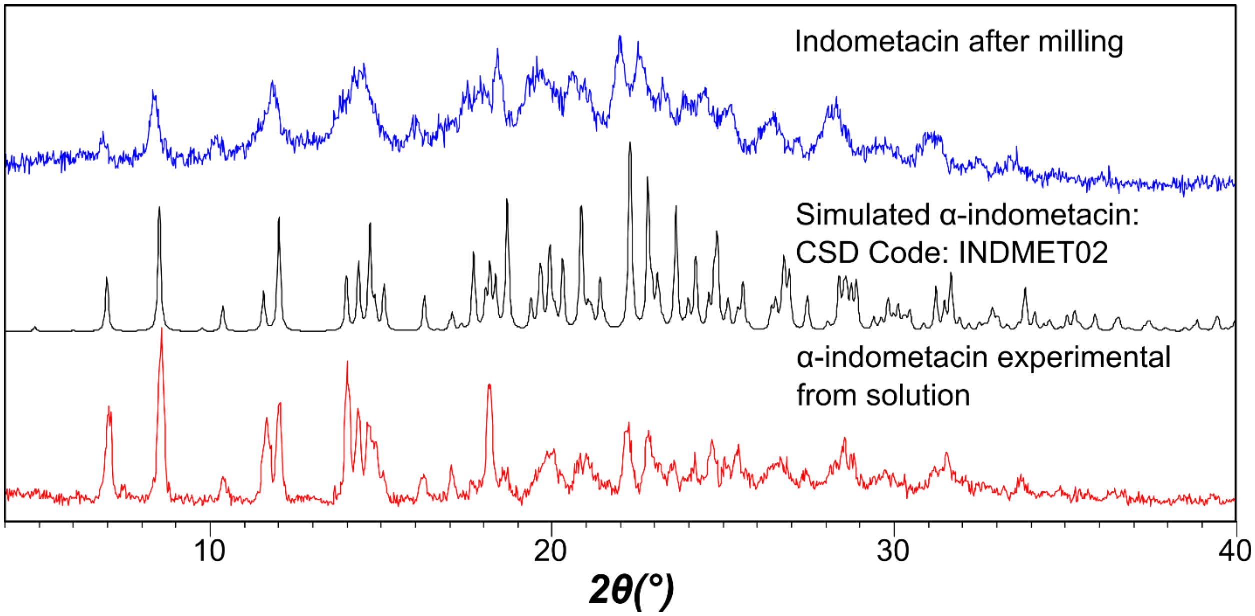

Figure S2. The XRPD pattern of a sample of solid **ind** after milling (top) compared with the patterns simulated for the reported structure of **α-ind** (middle) and measured for **α-ind** made from solution (bottom).

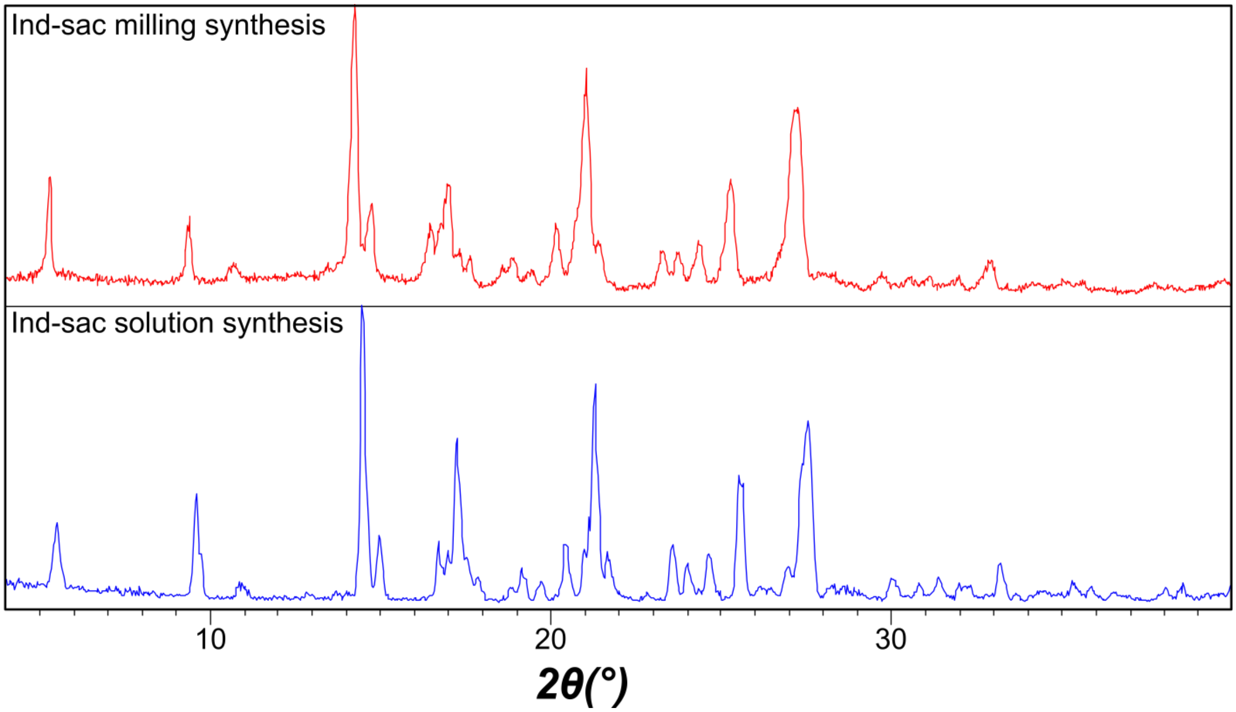

Figure S3. Overlay of the XRPD patterns of **ind-sac** cocrystal after milling (top) and **ind-sac** cocrystal synthesised from solution (bottom).

2. Experimental Setup

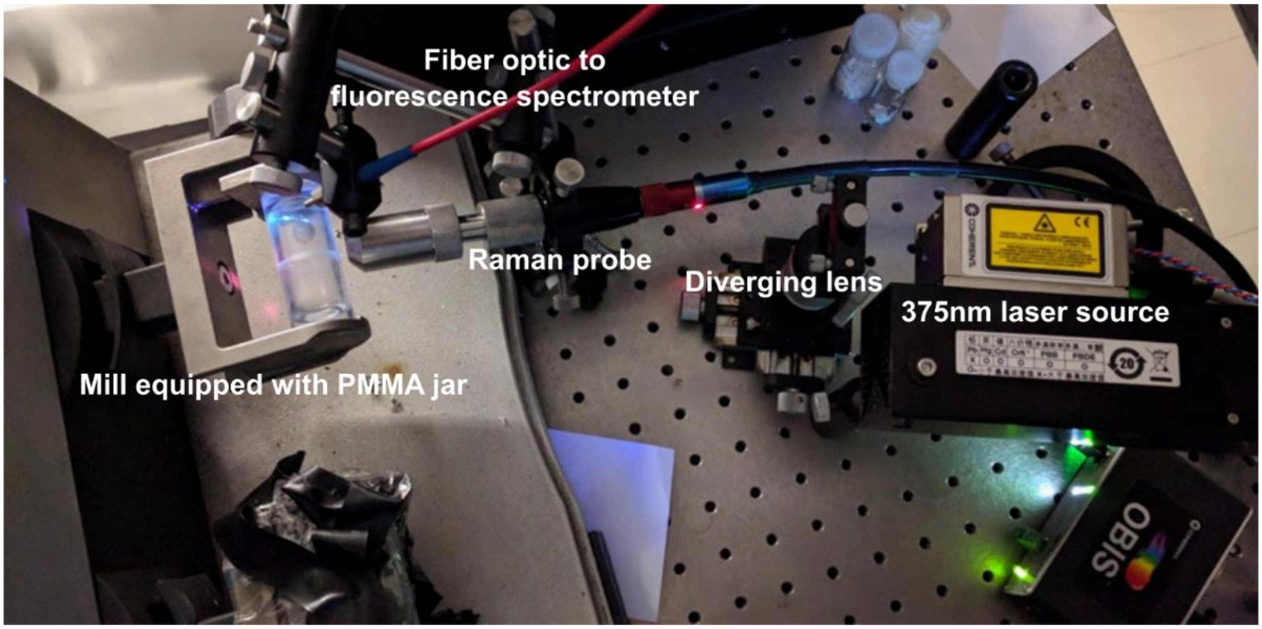

Figure S4. Picture of the experimental setup, showing an empty 15 mL volume poly(methyl methacrylate) (PMMA) jar mounted on a milling station of the Retsch™ MM400 mixer mill, loaded with a zirconia milling ball. The bottom probe is the Raman spectroscopy probe of a Kaiser Optics RXN1 system, while the blue and red fiber optic connects to the QE65000 spectrometer used for fluorescence measurements. A 375nm OBIS laser source and diverging lens provide the excitation source for fluorescence measurements.

3. Fluorescence Excitation and Emission Spectra and Lifetime Measurements

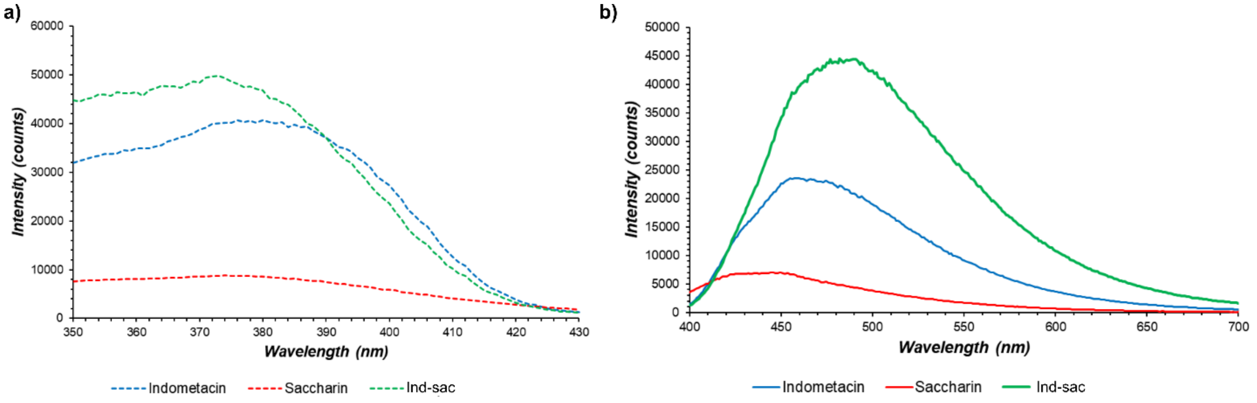

Figure S5. a) Excitation spectra monitored at 450 nm of **γ-ind** (blue), **sac** (red) and **ind-sac** (green); b) fluorescence emission spectra of solid **γ-ind**, **sac**, and **ind-sac**, under excitation at 380 nm. All spectra were recorded on a well-plate reader.

Table S1. Lifetimes of γ-indometacin, saccharin, and ind-sac cocrystal.

Compound	Fluorescence Lifetime <τ> (ns)	Chi Squared χ2
**γ-ind**	1.78	1.032
**sac**	4.29	4.56
**ind-sac**	5.62	1.17

4. Band-Gap Measurements

Table S2. Band gaps (in eV) for the different crystalline phases, measured by UV-Vis spectroscopy.

Phase	Measured band gap / eV
**ind-sac** cocrystal	2.95(2)
***γ*-ind**	2.95(3)
***α*-ind**	2.88(2)
***am*-ind**	2.56(1)

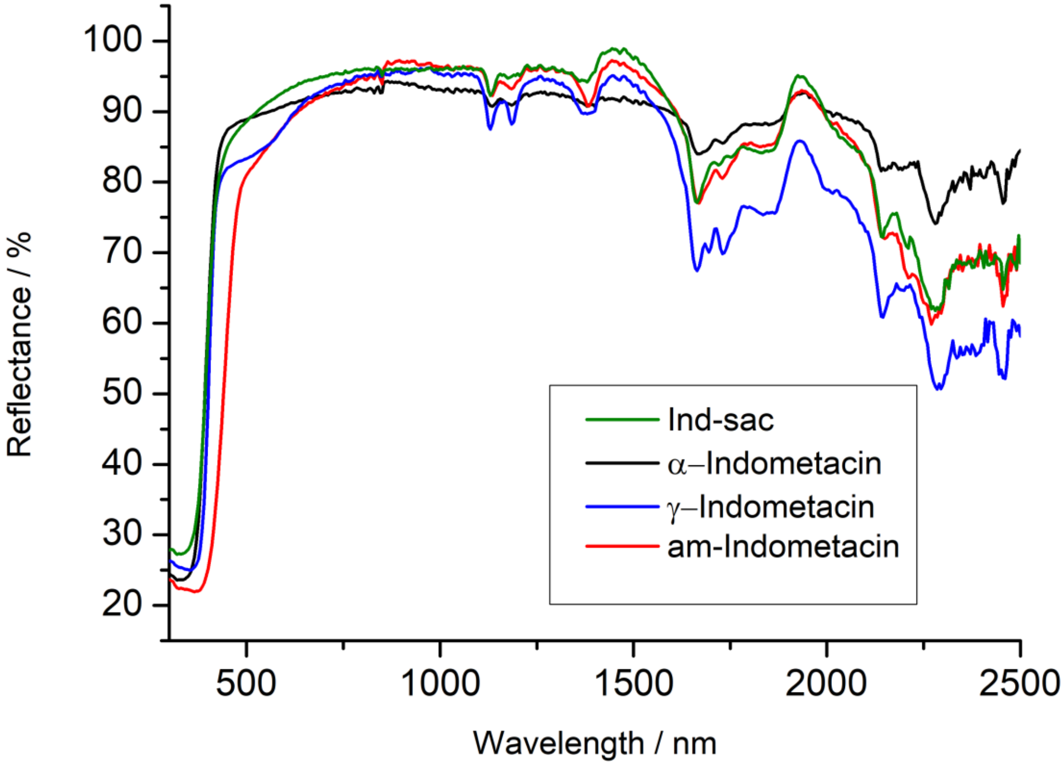

Figure S6. Overlay of full UV-Vis spectra for the **ind-sac** cocrystal (green line), **α-ind** (black line), **γ-ind** (blue line), and **am-ind** (red line).

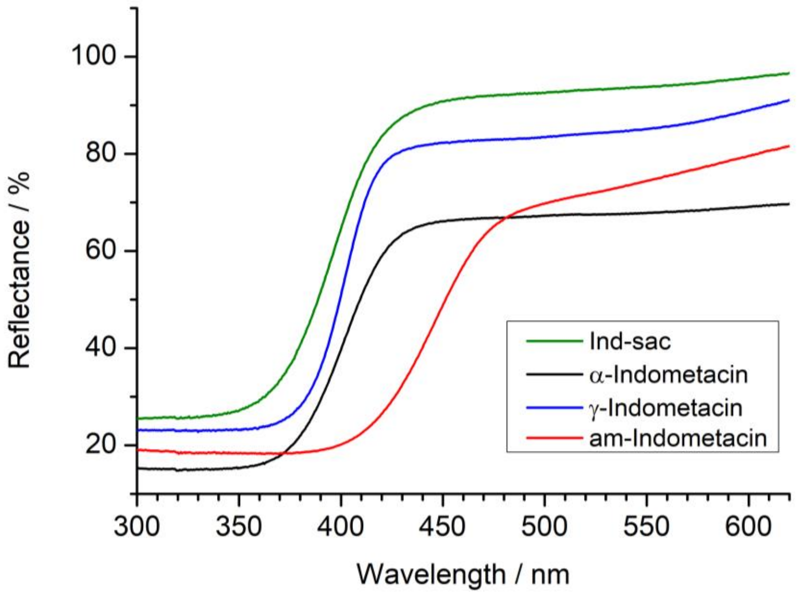

Figure S7. Overlay of UV-Vis spectra for the **ind-sac** cocrystal (green line), **α-ind** (black line), **γ-ind** (blue line), and **am-ind** (red line), used to calculate the respective band gaps.

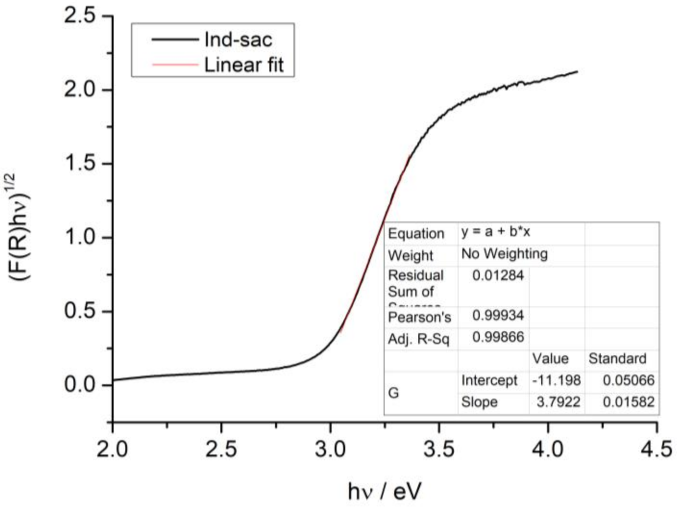

Figure S8. Linear fit of the UV-Vis spectrum of solid **ind-sac** used for band gap determination.

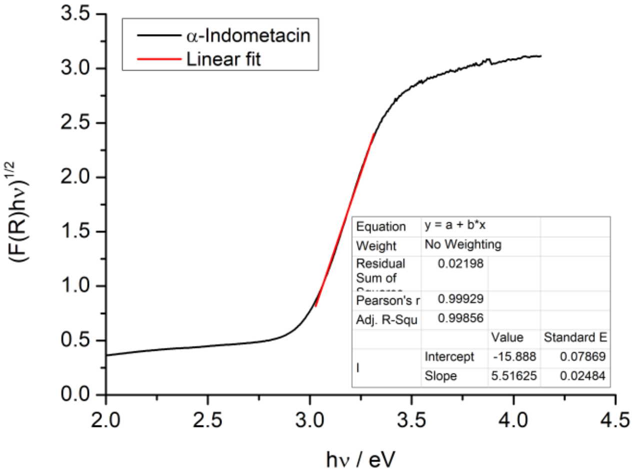

Figure S9. Linear fit of the UV-Vis spectrum of solid **α-ind** used for band gap determination.

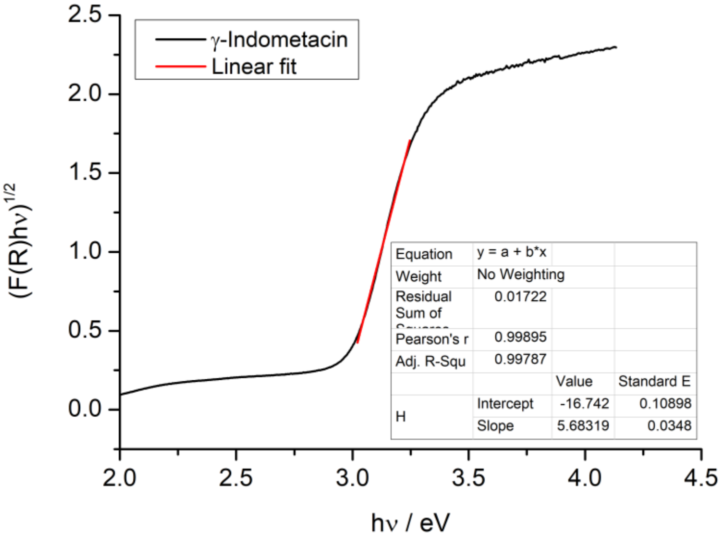

Figure S10. Linear fit of the UV-Vis spectrum of solid **γ-ind** used for band gap determination.

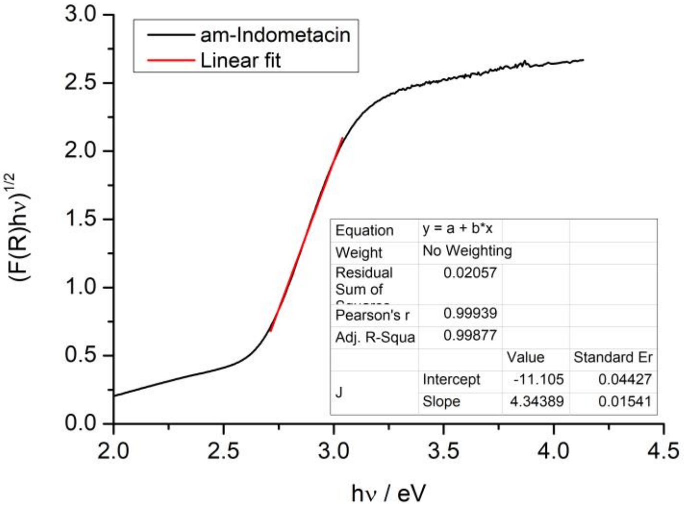

Figure S11. Linear fit of the UV-Vis spectrum of solid **am-ind** used for band gap determination.

5. Raman Spectra of Starting Materials and Products

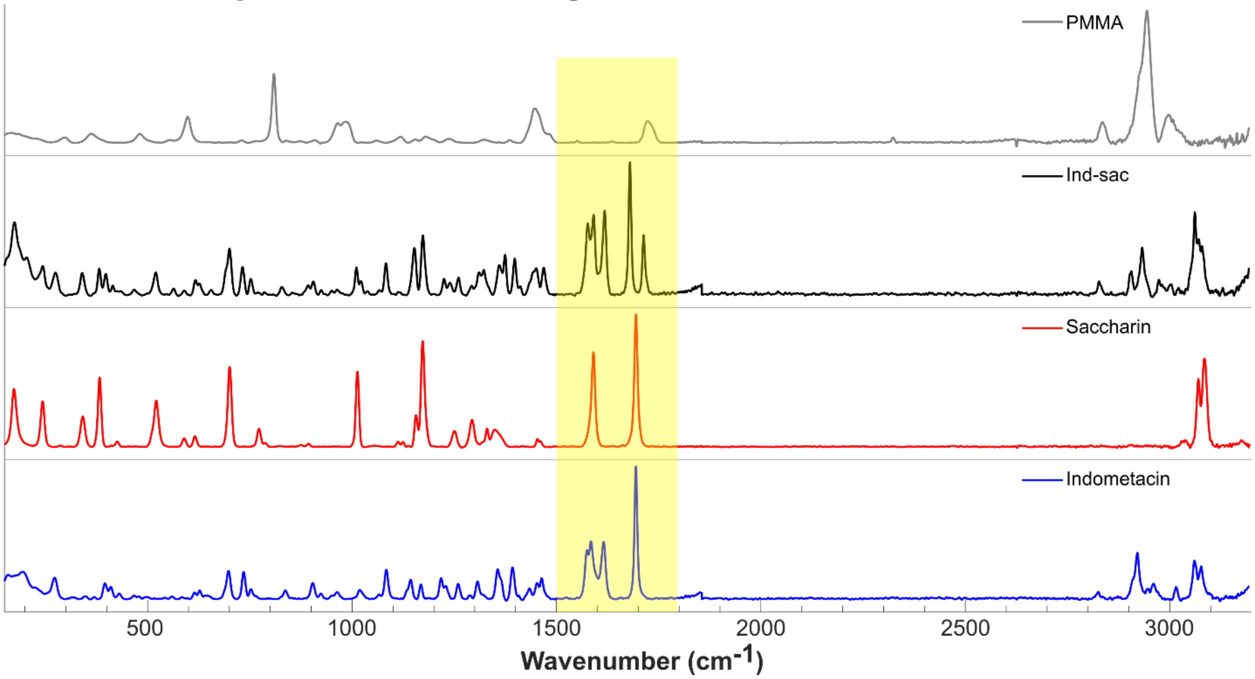

Figure S12. Comparison of baseline-corrected Raman spectra for solid samples of (bottom-to-top): **γ-ind**, **sac**, and **ind-sac** cocrystal along with the Raman spectrum of the PMMA milling jar which can interfere with the sample signal. The highlighted region between 1500-1800 cm^{-1} was used for reaction monitoring due to strong sample signals and minimal interference from PMMA.

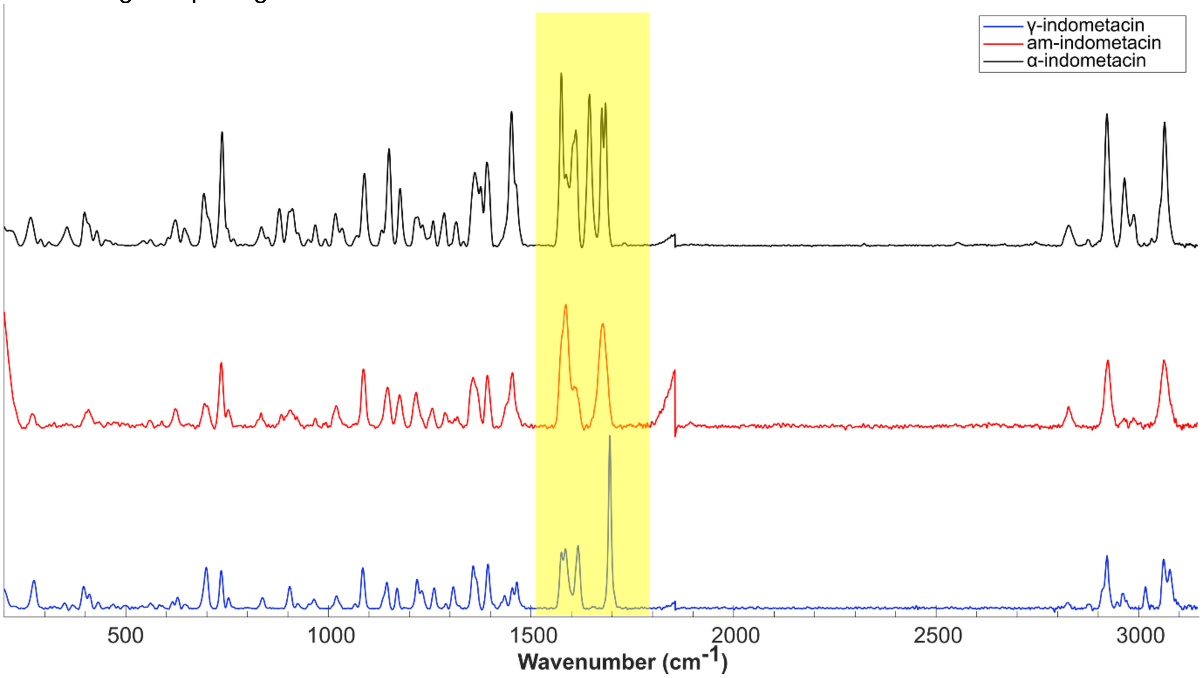

Figure S13. Baseline corrected Raman spectra for solid samples of (bottom-to-top): **γ-**, **α-**, and ***am*-ind**. The region between 1500-1800 cm^{-1} was used for reaction monitoring due to strong sample signals and minimal interference from the PMMA milling vessel.

6. Fitting of *in situ* spectroscopy datasets for ball-milling cocrystallisation of ind and sac

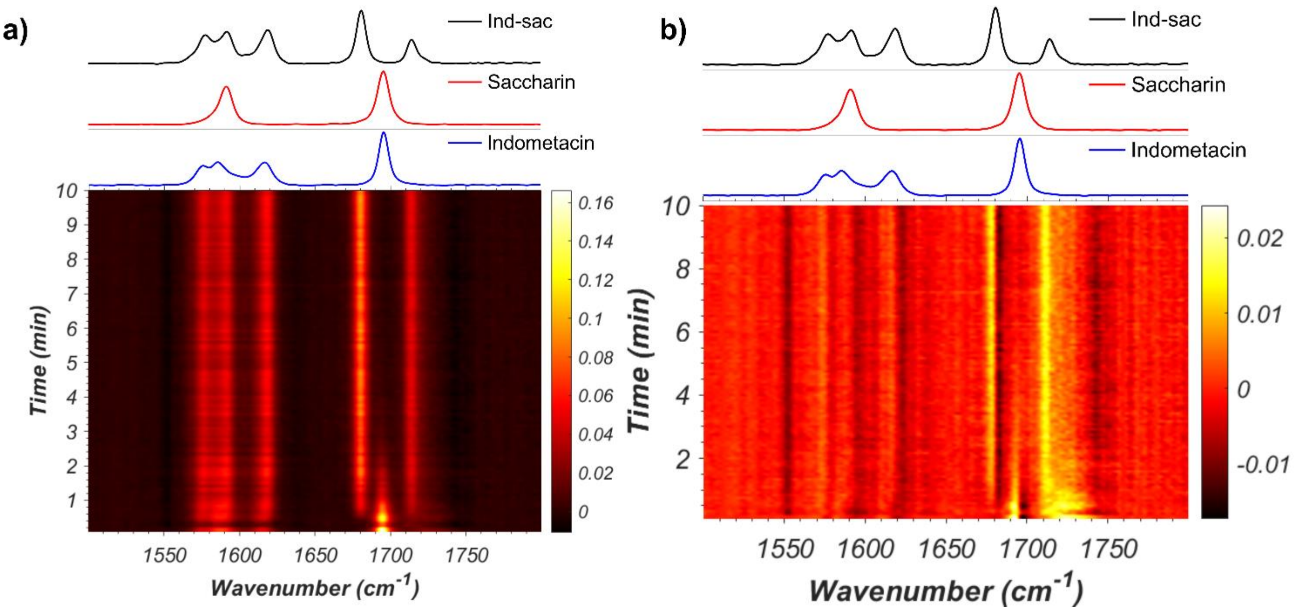

Figure S14. (a) (top) Raman spectra of solid **γ-ind**, **sac**, and **ind-sac** cocrystal and (bottom) normalised time-resolved Raman spectra acquired during the mechanochemical ball-milling synthesis of the **ind-sac** cocrystal. (b) (top) Raman spectra of solid **γ-ind**, **sac**, and the **ind-sac** cocrystal and (bottom) time-dependent residuals of the NNLS fitting.

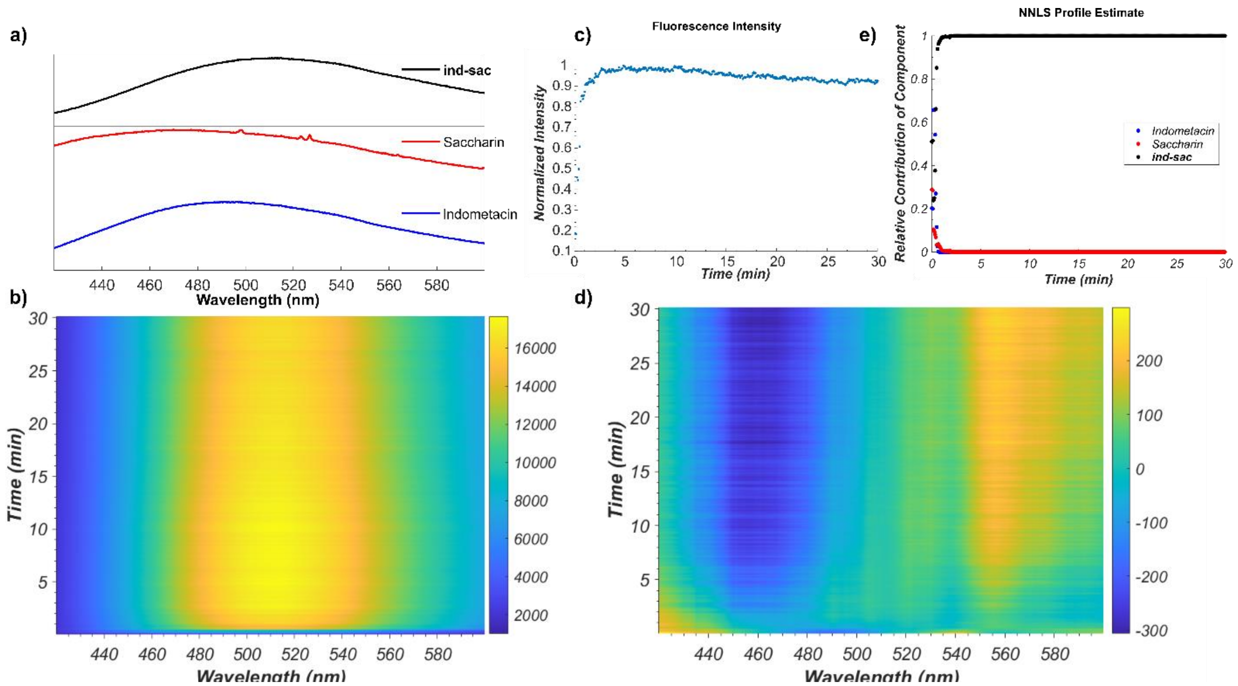

Figure S15. (a) Fluorescence emission behaviour of solid **γ-ind**, **sac**, and **ind-sac** samples. (b) Time-resolved fluorescence emission data acquired during the mechanochemical ball-milling synthesis of the **ind-sac** cocrystal. (c) Overall fluorescence intensity during the reaction normalised to the maximum emission intensity (d) Time-dependent residuals after NNLS fitting. (e) Relative amounts of **γ-ind**, **sac**, and the **ind-sac** cocrystal estimated using non-negative least squares fitting of the in situ measured dataset.

7. Solid-State ^{13}C NMR and FTIR-ATR Spectroscopy

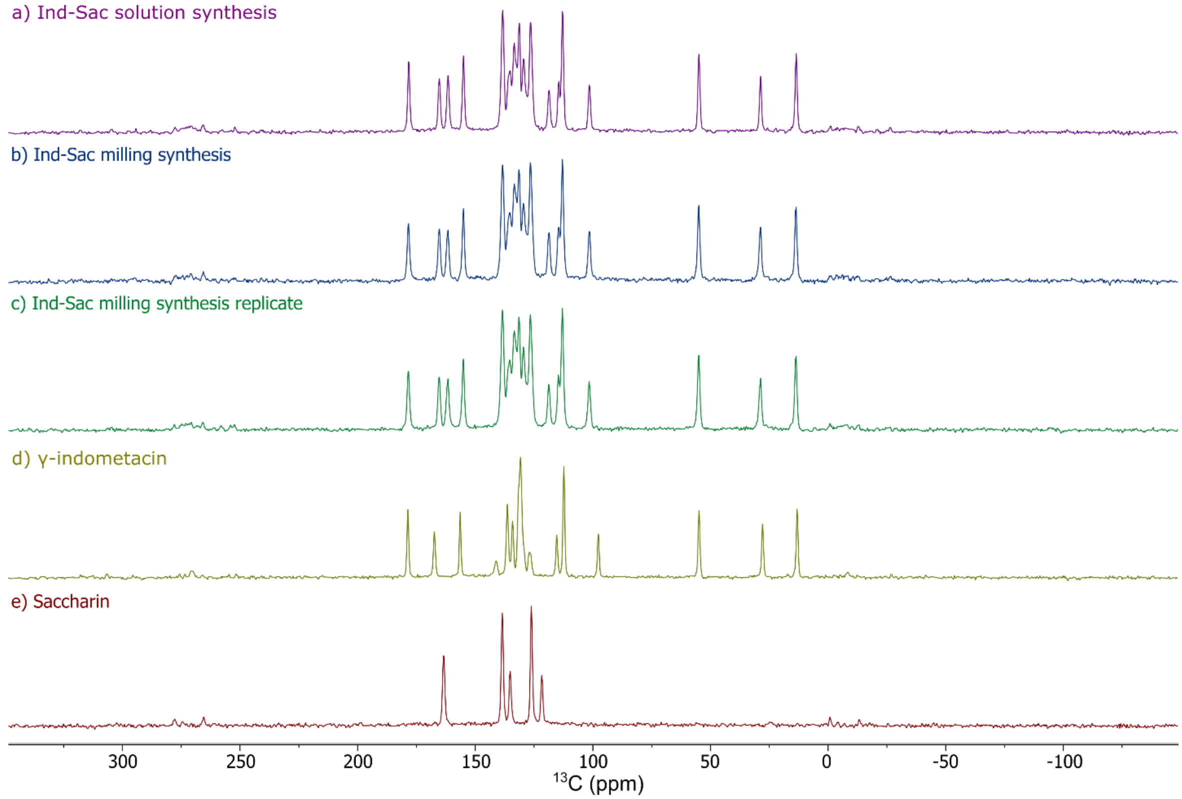

Figure S16. Overlay of ^{13}C CP-MAS-ssNMR spectra for: (a) the **ind-sac** cocrystal synthesised from solution and (b,c) by milling after real-time monitoring experiments, along with spectra of starting materials (d) **γ-ind** and (e) **sac**.

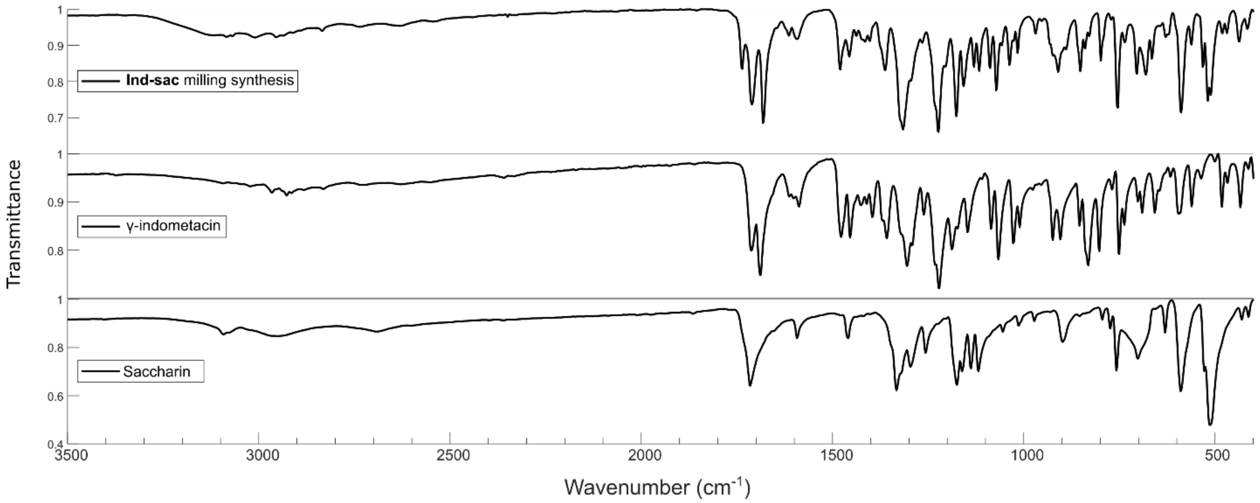

Figure S17. Fourier-transform infrared (FT-IR) spectra for (top-to-bottom): mechanochemically prepared **ind-sac** cocrystal (sample obtained after a real-time monitoring experiment), **γ-ind** and **sac**.

8. *In situ* Synchrotron Powder X-ray Diffraction Monitoring of Cocrystallisation by Milling

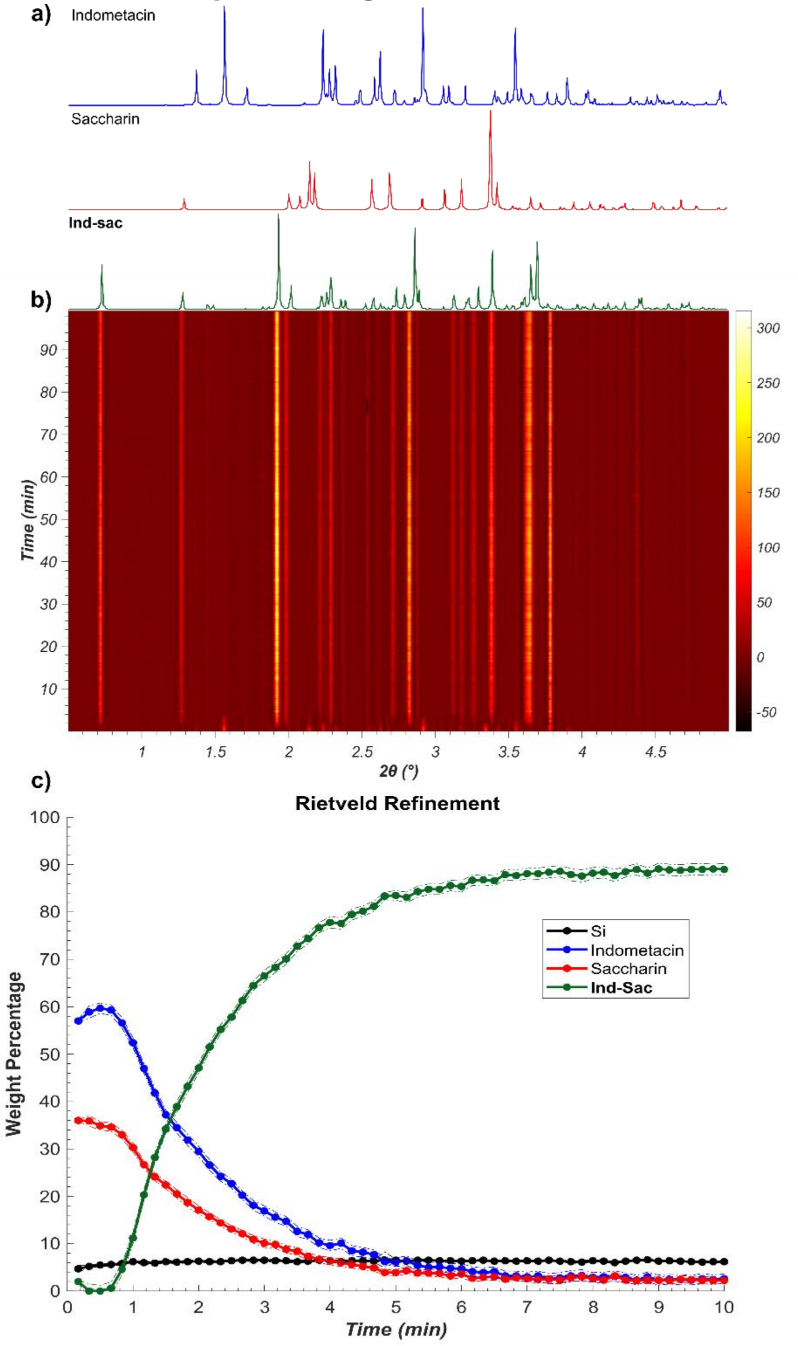

Figure S18. (a) Simulated XRPD patterns for **γ-ind** (CSD code INDMET), **sac** (CSD code SCCHRN03 and **ind-sac** cocrystal (CSD code UFERED). (b) Time-resolved synchrotron XRPD patterns showing the mechanochemical formation of **ind-sac,** in presence of crystalline silicon as an internal standard. (c) Weight percentage of each phase, as estimated by Rietveld refinement. The rate of conversion is slightly slower, than that observed by Raman and fluorescence emission measurements, likely due to the use of 7 mm steel milling balls.

9. Comparison of Predicted and Experimental Vibrational Spectra

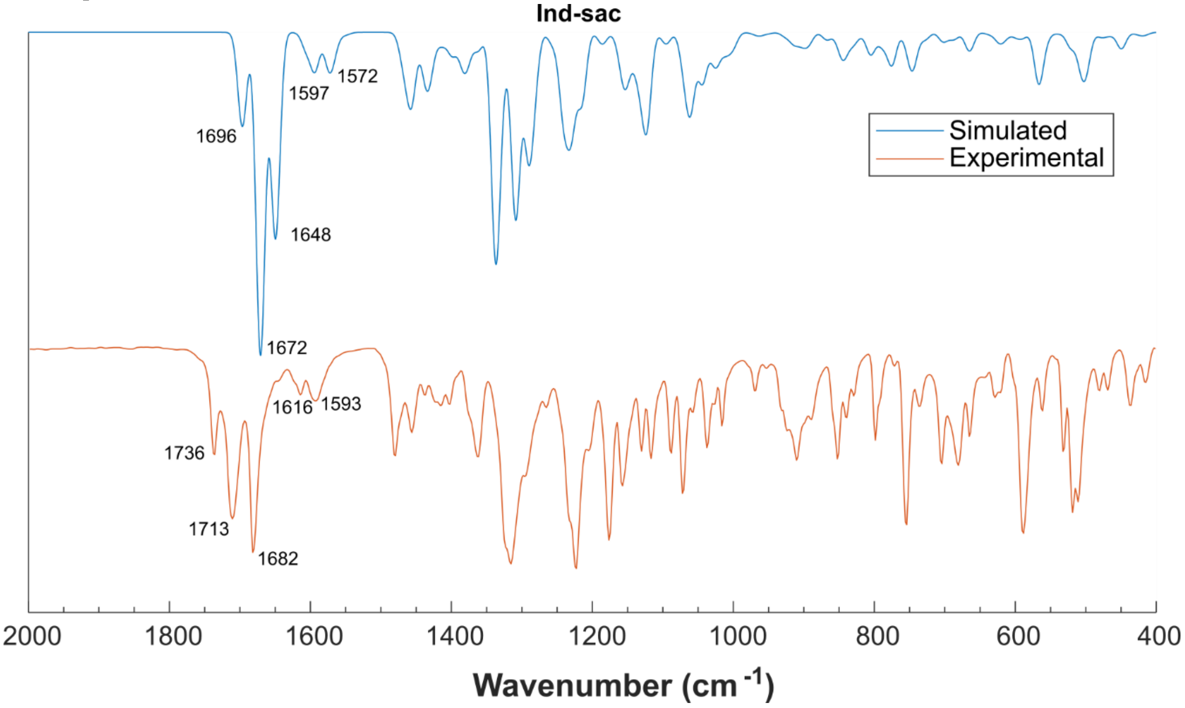

Figure S19. Comparison of the simulated (top) infrared spectrum obtained from periodic DFT calculations to the experimentally obtained (bottom) FTIR-ATR spectrum of the **ind-sac** cocrystal.

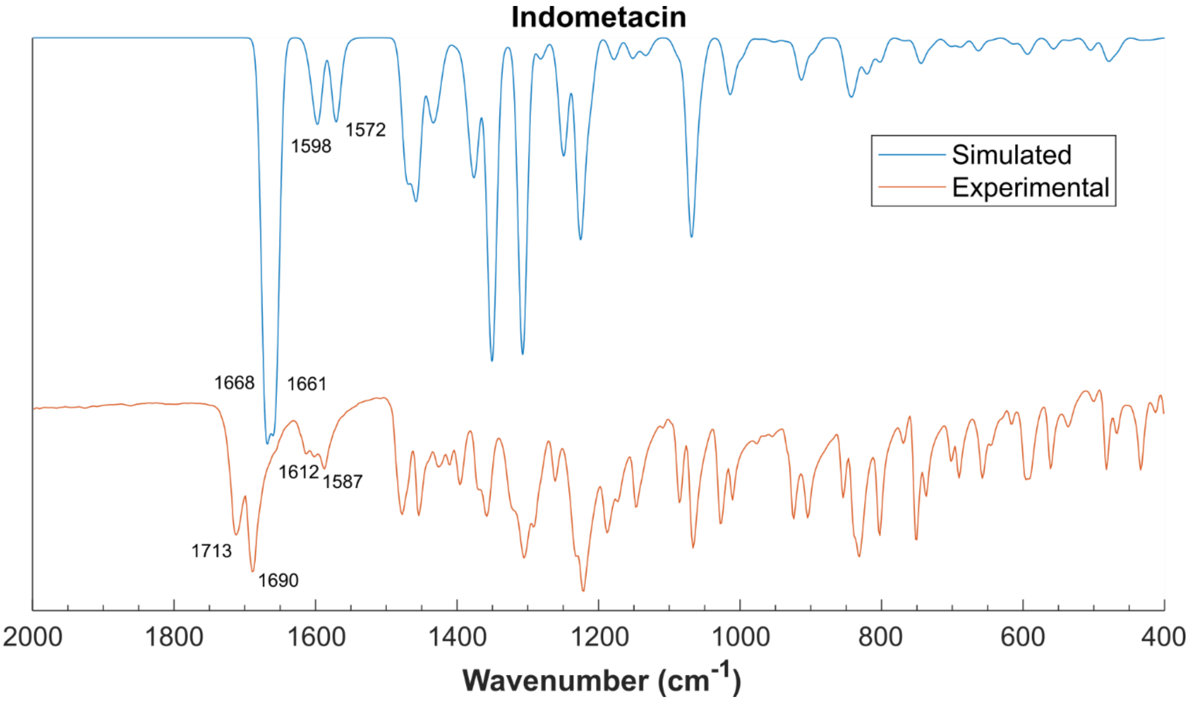

Figure S20. Comparison of the simulated (top) infrared spectrum obtained from periodic DFT calculations to the experimentally obtained (bottom) FTIR-ATR spectrum of **γ-ind**.

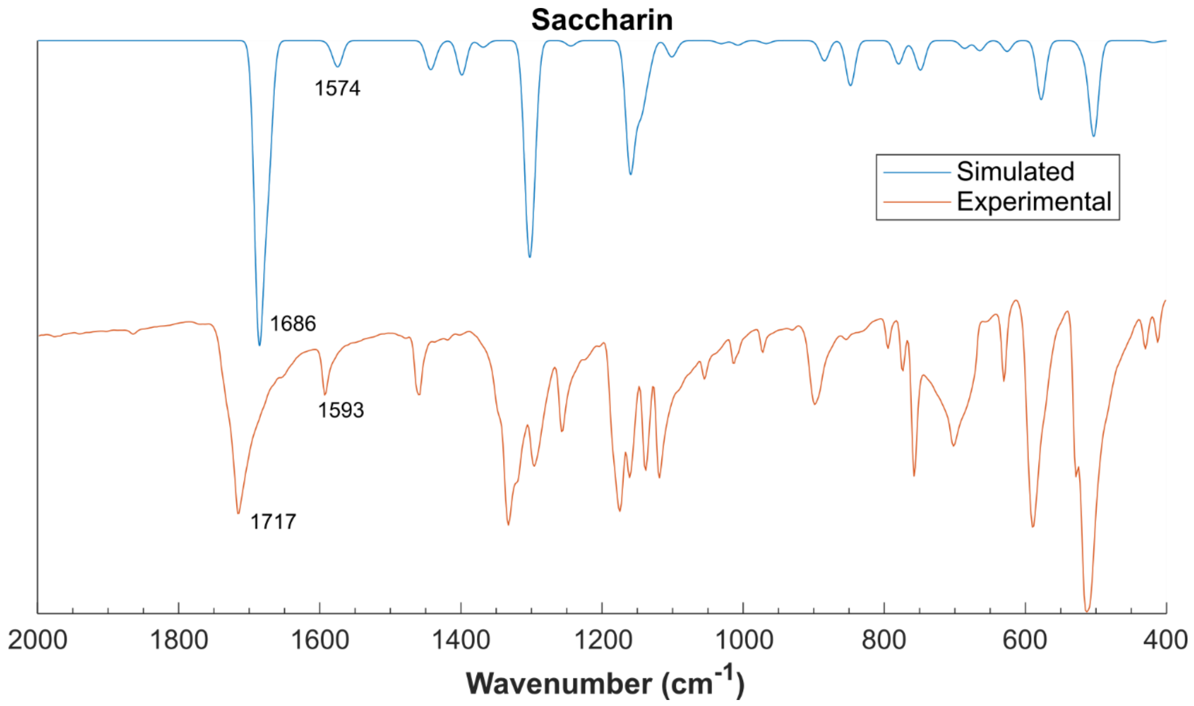

Figure S21. Comparison of the simulated (top) infared spectrum obtained from periodic DFT calculations to the experimentally obtained (bottom) FTIR-ATR spectrum of solid **sac**.

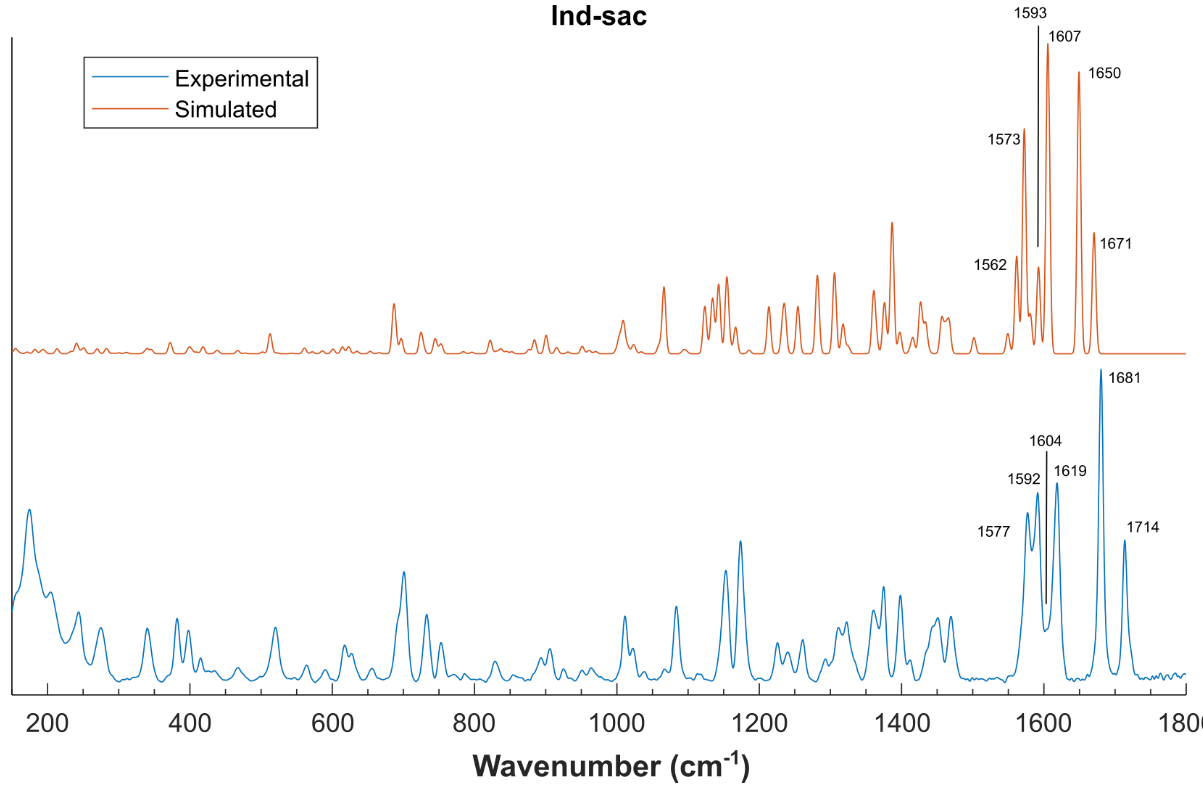

Figure S22. Comparison of the simulated (top) Raman spectrum obtained from periodic DFT calculations with the experimentally obtained (bottom) spectrum of the **ind-sac** cocrystal.

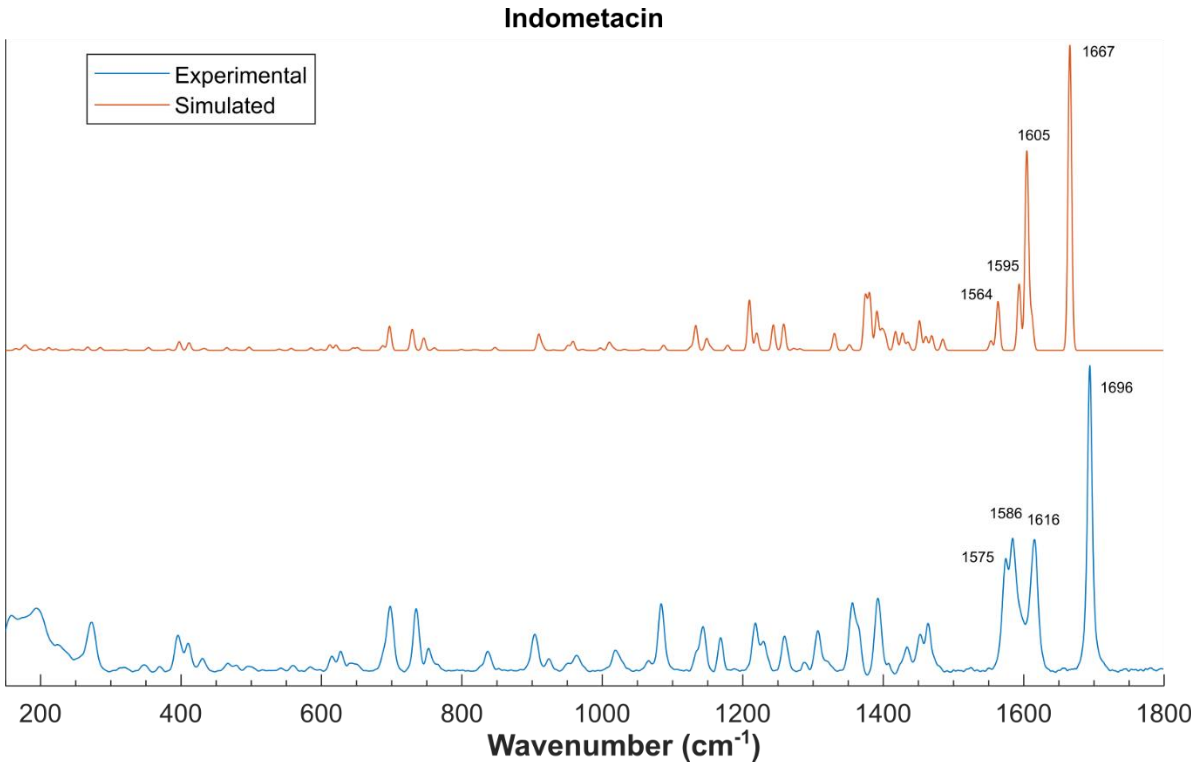

Figure S23. Comparison of the simulated (top) Raman spectrum obtained from periodic DFT calculations with the experimentally obtained (bottom) spectrum of **γ-ind**.

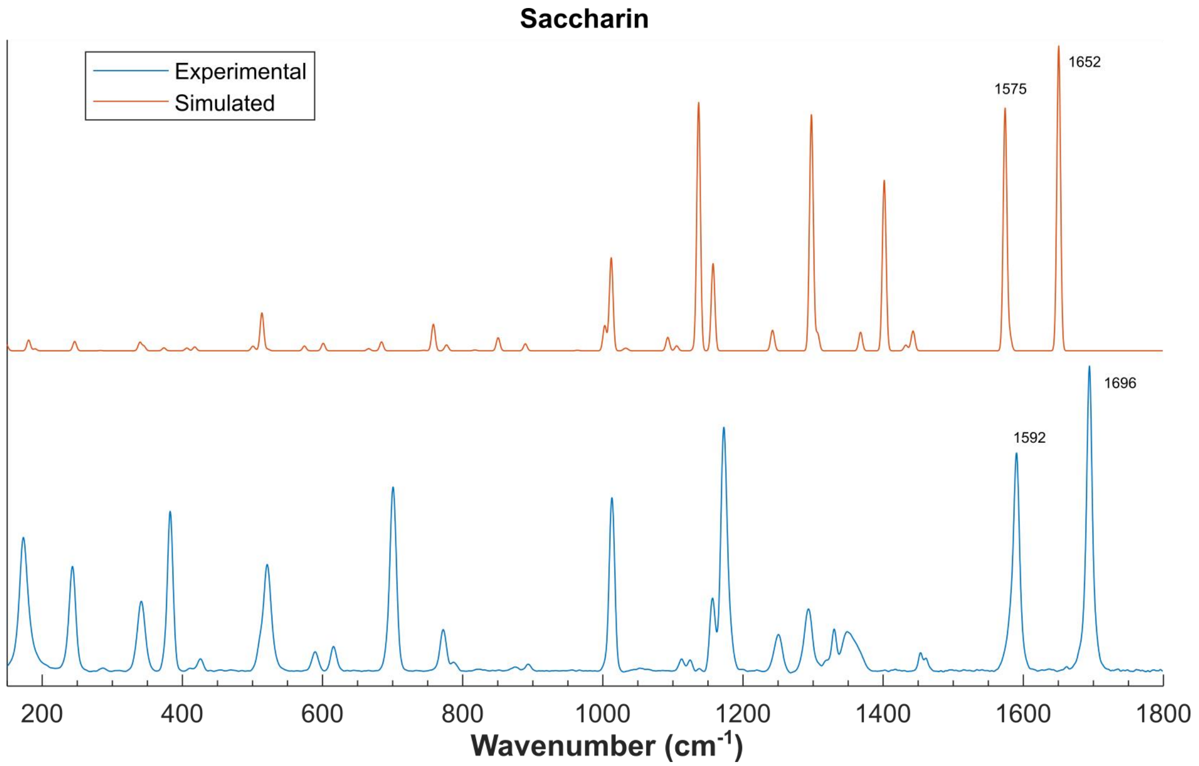

Figure S24. Comparison of the simulated (top) Raman spectrum obtained from periodic DFT calculations with the experimentally obtained (bottom) spectrum of solid **sac**.

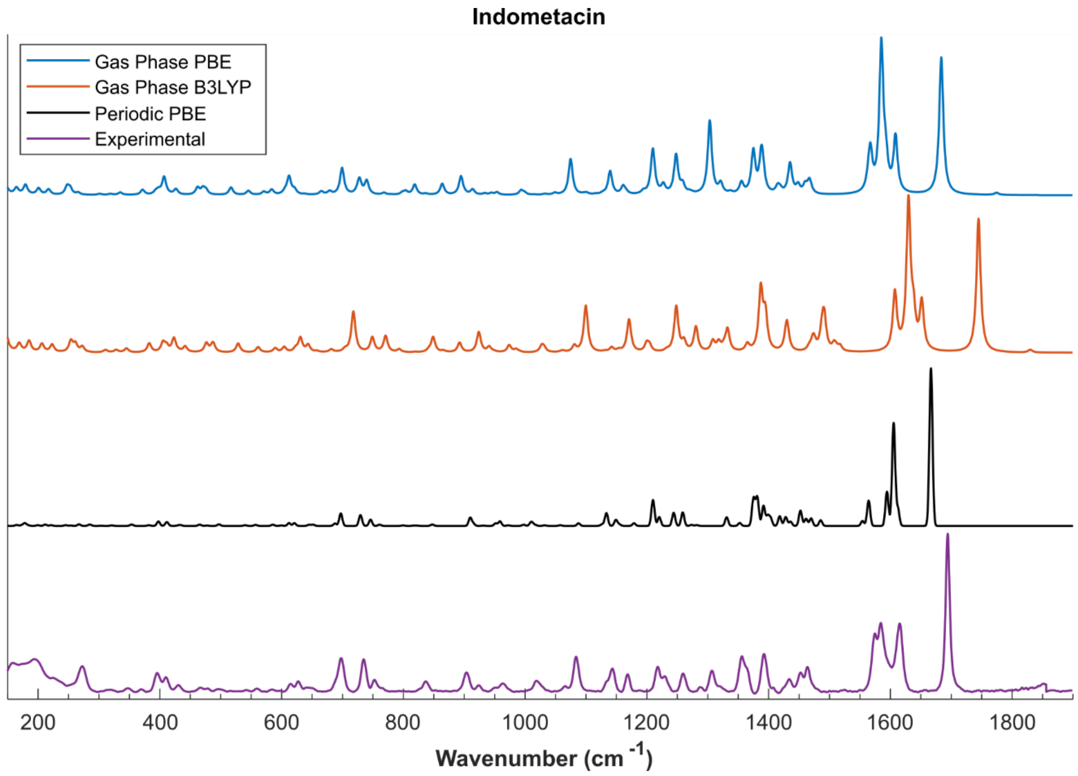

Figure S25. Comparison of experimental and calculated Raman spectra for **ind** (top-to-bottom): calculated in the gas phase using the PBE functional, calculated in the gas phase using the B3LYP functional, calculated for **γ-ind** using periodic DFT with the PBE-generalised gradient approximation functional and experimentally measured for **γ-ind**.

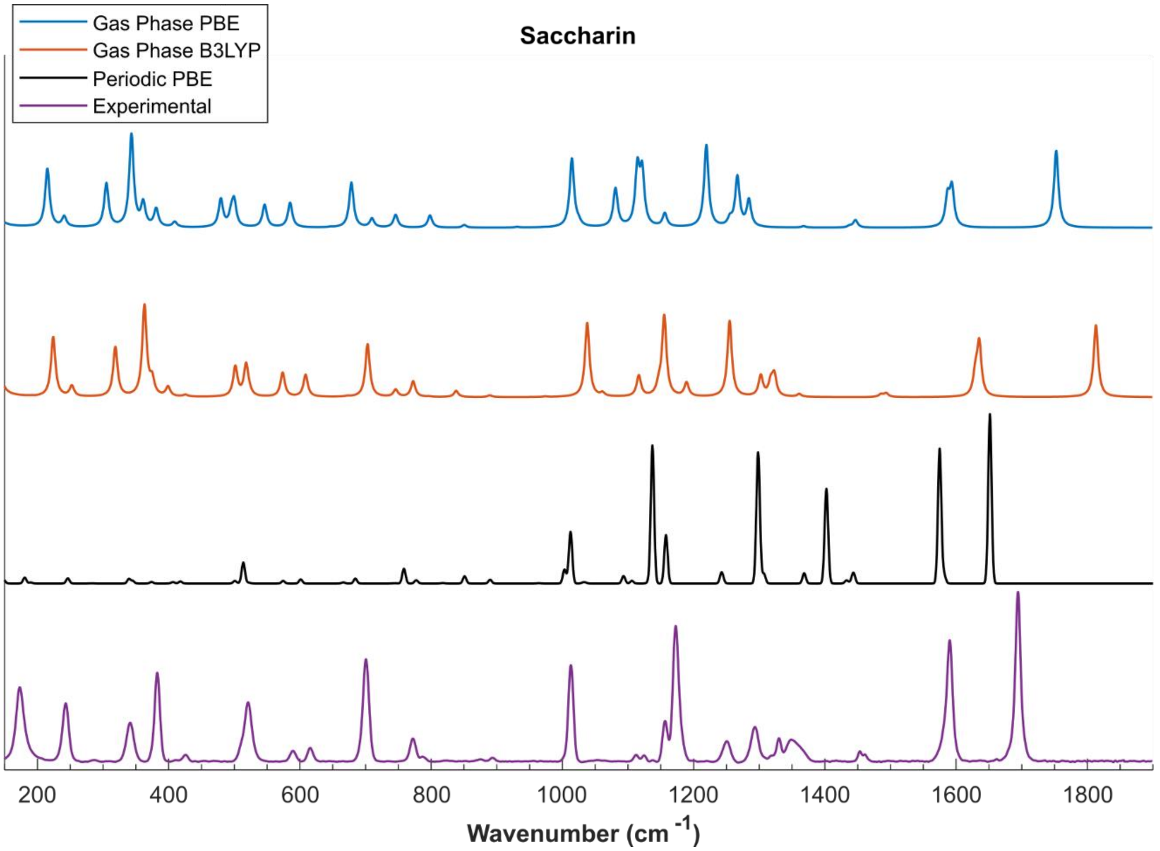

Figure S26. Comparison of experimental and calculated Raman spectra for **sac** (top-to-bottom): calculated in the gas phase using the PBE functional, calculated in the gas phase using the B3LYP functional, calculated for **sac** solid using periodic DFT with the PBE-generalised gradient approximation functional and experimentally measured for **sac** solid.

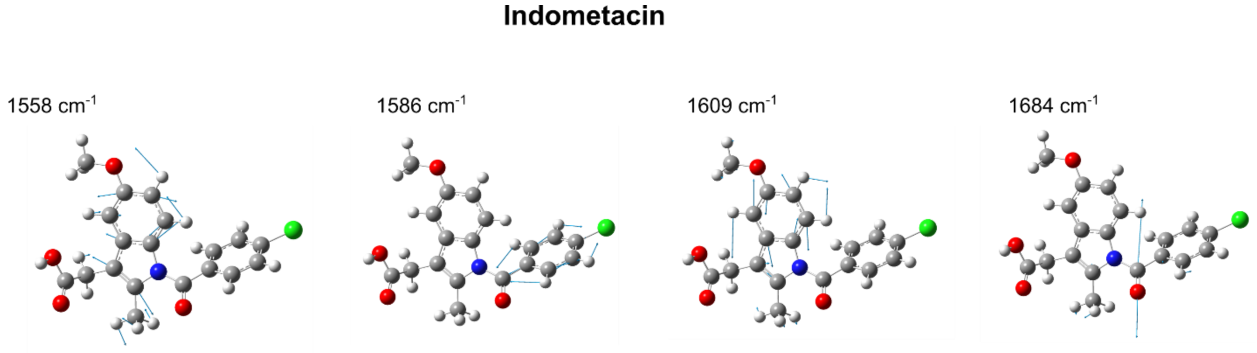

Figure S27. Key Raman-active vibrational modes of **ind** in the 1500-1800 cm^{-1} region used for *in situ* monitoring of the reaction as determined by gas phase DFT calculations using the hybrid functional PBE0. The amplitude of displacement vectors has been increased for visual clarity.

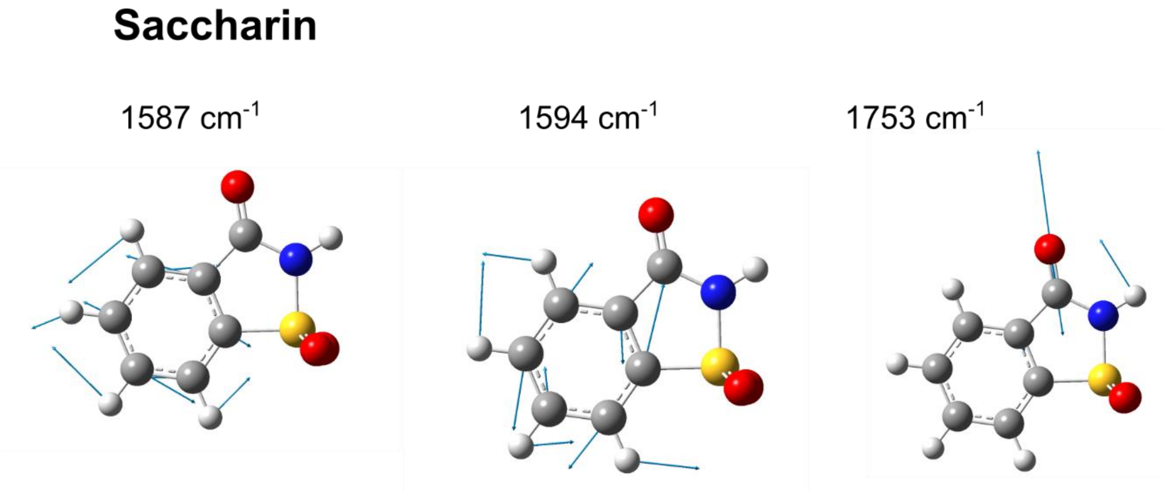

Figure S28. Key Raman-active vibrational modes of **sac** in the 1500-1800 cm^{-1} region used for *in situ* monitoring of the reaction as determined by gas phase DFT calculations using the hybrid functional PBE0. The amplitude of displacement vectors has been increased for visual clarity.

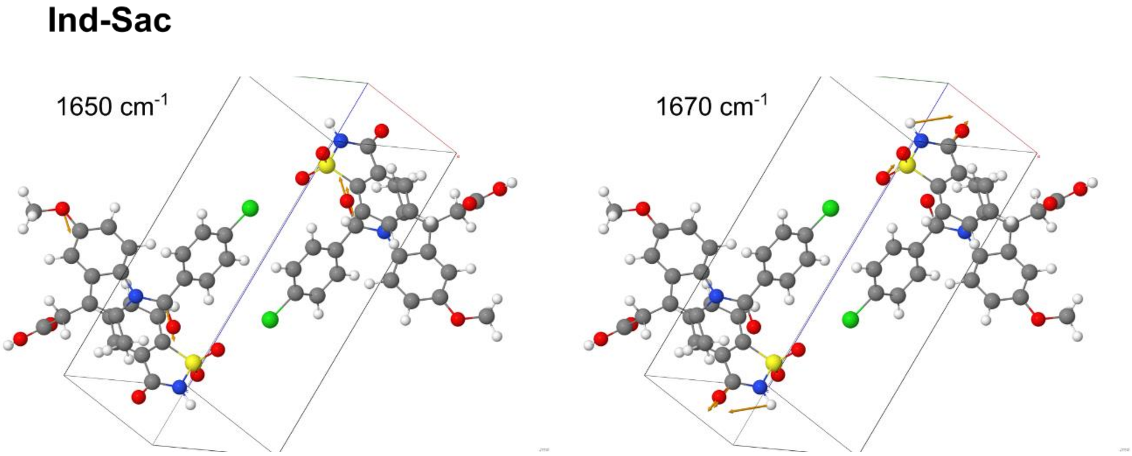

Figure S29. Key Raman-active vibrational modes of **ind-sac** which indicate the vibration at 1670 cm^{-1} arising from the **sac** carbonyl moiety stretch and the vibration at 1650 cm^{-1} arising from the **ind** carbonyl moiety stretch, as determined by periodic DFT calculations using the PBE functional. The amplitude of displacement vectors has been increased for visual clarity.

10. Comparison of Predicted and Experimental NMR Spectra

Table S3. Comparison of ^{13}C CP-MAS-SSNMR chemical shift values (ppm) determined experimentally to those predicted from periodic DFT calculations, listed from highest to lowest chemical shift.

saccharin			γ-indometacin			ind-sac		
Experiment	Predicted	Difference (Pred-Exp)	Experiment	Predicted	Difference (Pred-Exp)	Experiment	Predicted	Difference (Pred-Exp)
163.45	163.67	0.22	178.66	185.46	6.80	178.43	183.55	5.12
138.51	143.17	4.66	167.38	169.48	2.10	165.35	165.78	0.43
138.51	141.19	2.68	156.43	159.11	2.68	161.72	163.59	1.87
135.12	138.03	2.91	141.11	148.54	7.43	155.1	158.43	3.33
126.13	129.19	3.06	136.37	139.37	3.00	138.49	145.19	6.70
126.13	127.49	1.36	134.15	136.01	1.86	138.49	142.96	4.47
121.71	123.7	1.99	134.15	134.89	0.74	138.49	141.54	3.05
			131.75	134.83	3.08	135.57	139.89	4.32
			130.8	133.16	2.36	133.47	137.82	4.35
			130.8	132.76	1.96	133.47	136.3	2.83
			126.91	131.04	4.13	131.46	135.01	3.55
			126.26	128.51	2.25	131.46	134.43	2.97
			115.36	116.73	1.37	131.46	133.23	1.77
			112.35	116.46	4.11	129.43	131.73	2.30
			112.35	111.94	-0.41	129.43	130.07	0.64
			97.68	97.11	-0.57	126.57	128.76	2.19
			54.88	56.05	1.17	126.57	128.74	2.17
			27.89	27.79	-0.10	126.57	127.83	1.26
			13.2	14.11	0.91	118.77	120.63	1.86
						114.68	117.09	2.41
						112.99	116.13	3.14
						112.99	113.24	0.25
						101.57	101.76	0.19
						55.01	56.18	1.17
						28.82	28.95	0.13
						13.67	13.08	-0.59

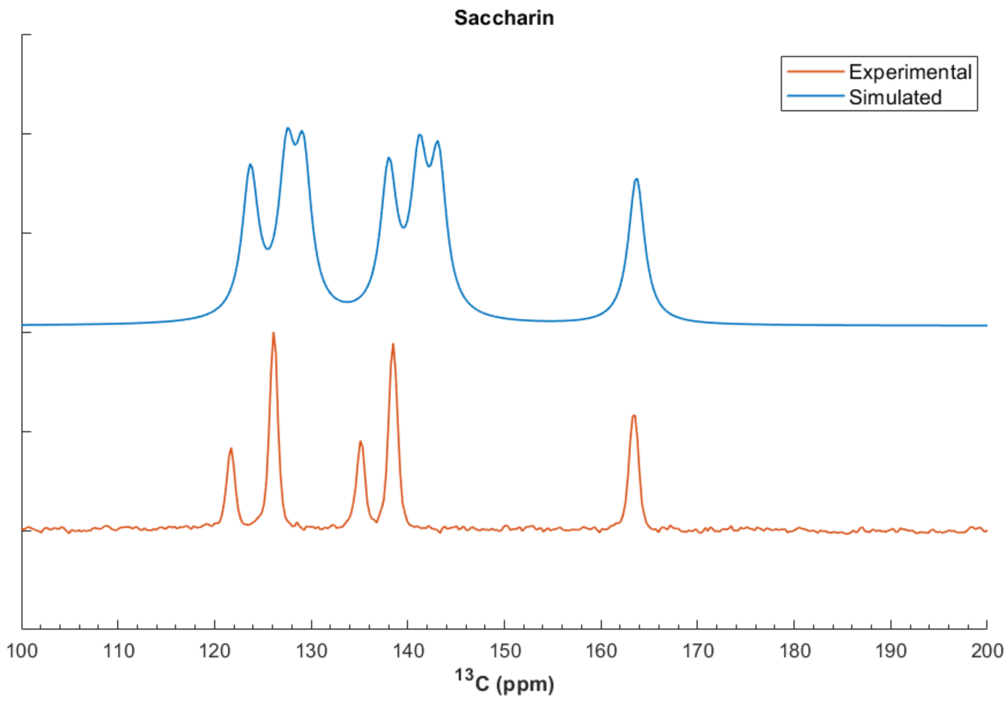

Figure S30. Comparison of the simulated ^{13}C NMR spectrum (top) obtained from periodic DFT calculations with the experimentally obtained (bottom) spectrum of solid **sac**.

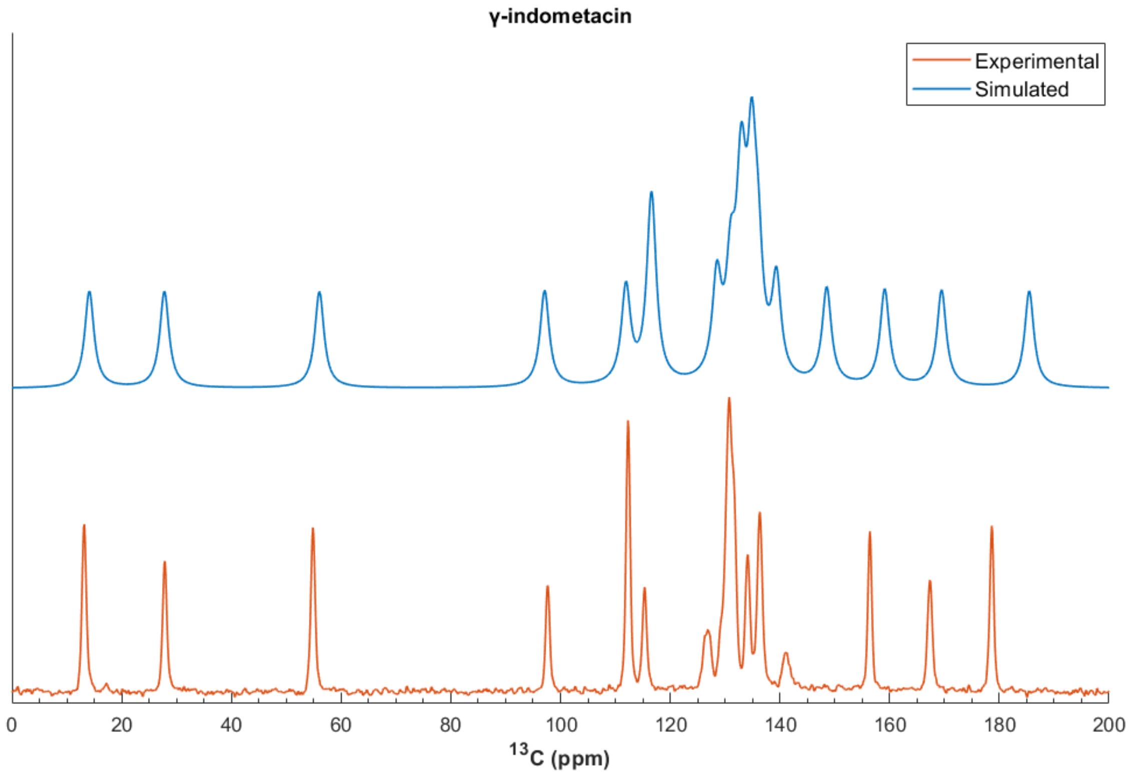

Figure S31. Comparison of the simulated ^{13}C NMR spectrum (top) obtained from periodic DFT calculations with the experimentally obtained (bottom) spectrum of **γ-ind**.

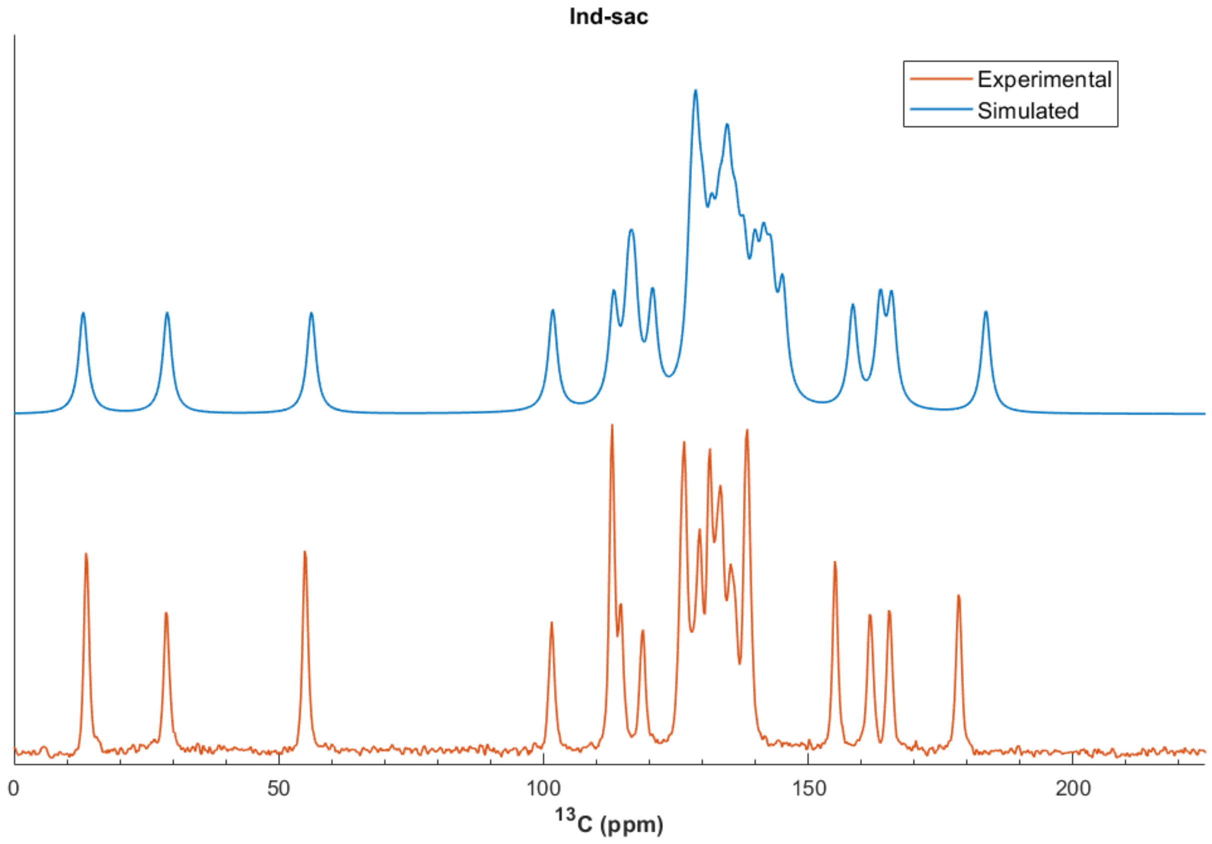

Figure S32. Comparison of the simulated ^{13}C NMR spectrum (top) obtained from periodic DFT calculations with the experimentally obtained (bottom) spectrum of the **ind-sac** cocrystal.

Table S4. Predicted ^{13}C CP-MAS-SSNMR chemical shift values from periodic DFT calculations for each carbon in the starting materials (solid **sac** and **γ-ind**), as well as the product **ind-sac** cocrystal. Carbon atoms with significant differences between starting material and cocrystal are highlighted in red.

	atom	shielding / ppm starting materials	shielding / ppm cocrystal	chemical shift / ppm starting materials	chemical shift / ppm cocrystal	Absolute difference /ppm
saccharin	C1	43.13	42.79	127.49	127.83	0.34
	C2	41.43	41.88	129.19	128.74	0.45
	C3	32.59	32.8	138.03	137.82	0.21
	C4	27.45	30.73	143.17	139.89	3.28
	C5	46.92	49.99	123.7	120.63	3.07
	C6	29.43	29.08	141.19	141.54	0.35
	C7	6.95	7.03	163.67	163.59	0.08
indomethacin	C8	39.58	40.55	131.04	130.07	0.97
	C9	53.89	54.49	116.73	116.13	0.6
	C10	58.68	57.38	111.94	113.24	1.3
	C11	11.51	12.19	159.11	158.43	0.68
	C12	73.51	68.86	97.11	101.76	4.65
	C13	37.46	37.39	133.16	133.23	0.07
	C14	54.16	53.53	116.46	117.09	0.63
	C15	31.25	27.66	139.37	142.96	3.59
	C16	114.57	114.44	56.05	56.18	0.13
	C17	142.83	141.67	27.79	28.95	1.16
	C18	-14.84	-12.93	185.46	183.55	1.91
	C19	156.51	157.54	14.11	13.08	1.03
	C20	1.14	4.84	169.48	165.78	3.7
	C21	35.73	36.19	134.89	134.43	0.46
	C22	34.61	34.32	136.01	136.3	0.29
	C23	42.11	41.86	128.51	128.76	0.25
	C24	22.08	25.43	148.54	145.19	3.35
	C25	37.86	38.89	132.76	131.73	1.03
	C26	35.79	35.61	134.83	135.01	0.18

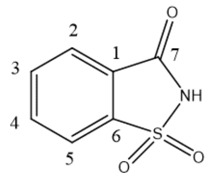

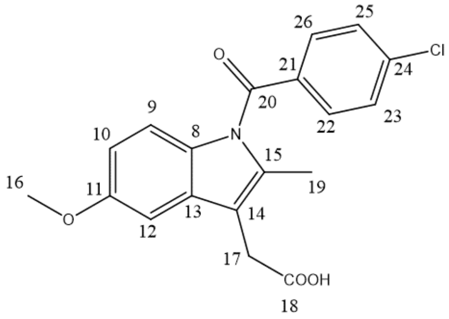

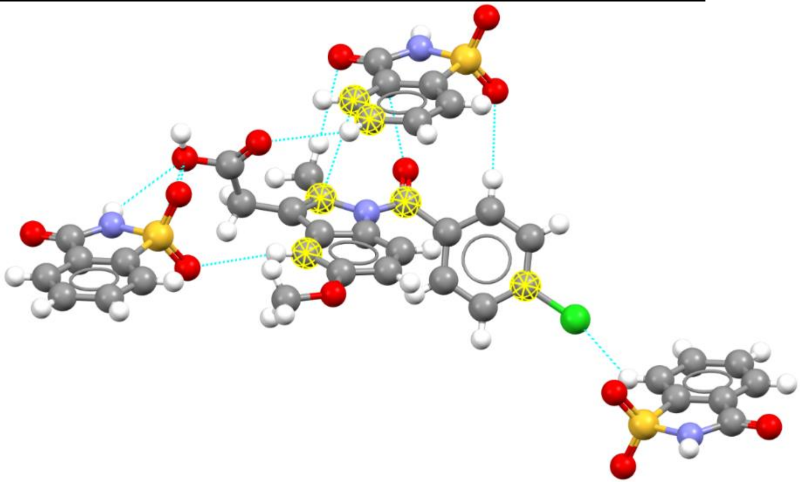

Figure S33. Visualisation of short contacts in the **ind-sac** cocrystal structure, with yellow highlights designating the carbon atoms exhibiting a difference in ^{13}C SS-NMR shifts of more than 3 ppm between the respective **sac** and **γ-ind** starting materials and the **ind-sac** cocrystal, as calculated by periodic DFT.

11. TD-DFT Orbital Transitions

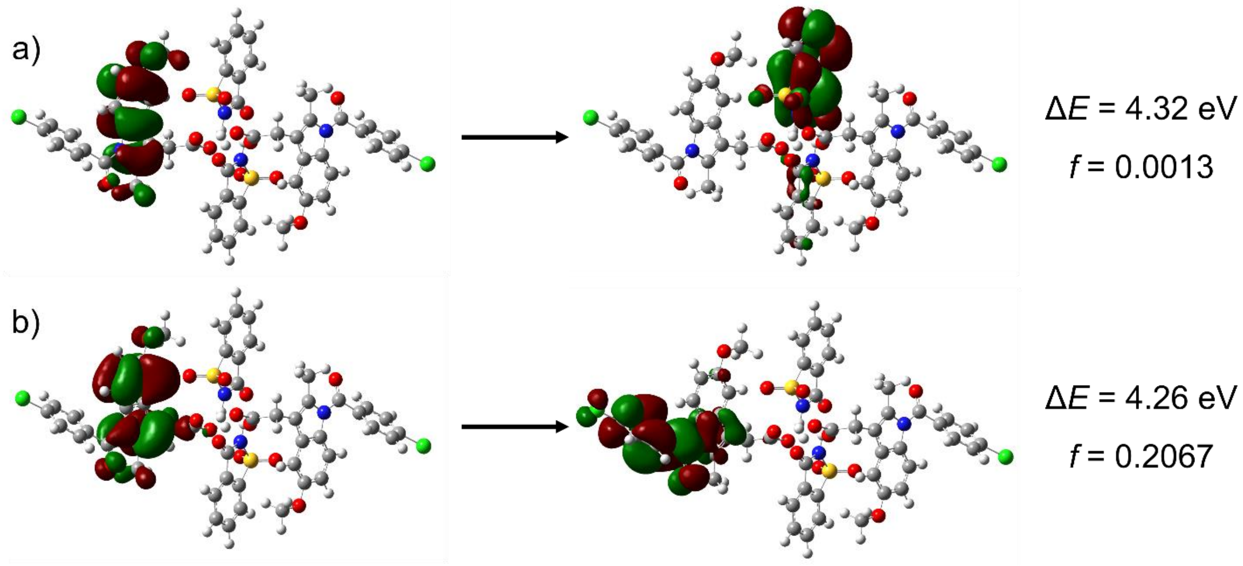

Figure S34. Molecular TD-DFT calculations using range-separated CAM-B3LYP functional on a cluster containing two **ind** and two **sac** molecules show that charge transfer state (a) has a very low oscillator strength, whereas the electronic transition localised on the **ind** molecule (b) is two orders of magnitude more intense.

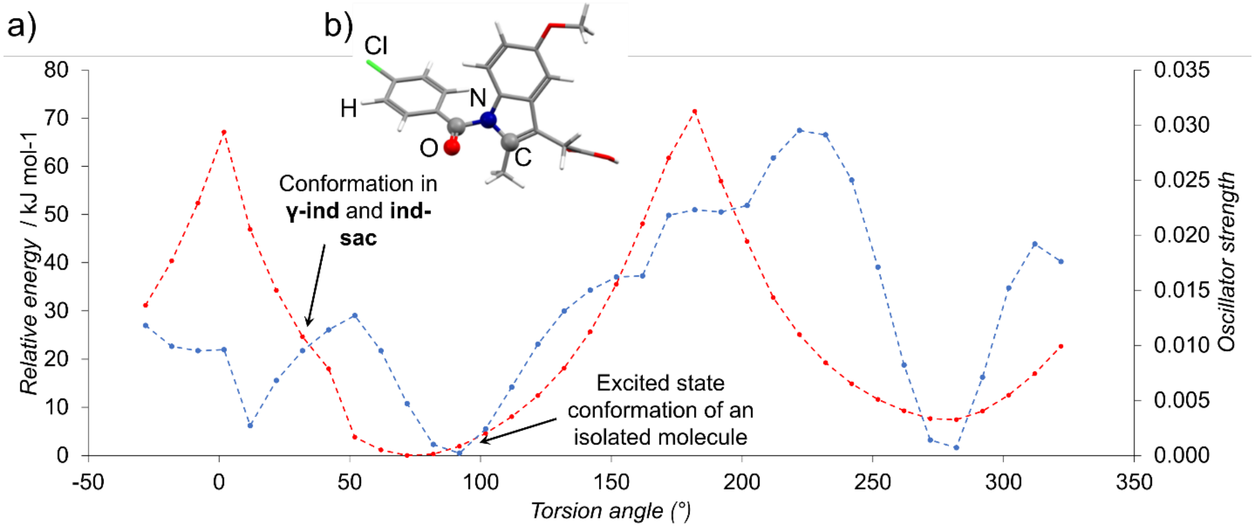

Figure S35. Calculated variation of the total energy (red) and oscillator strength (blue) for the S1 excited state of an **ind** molecule as a function of the orientation of the carbonyl group (the corresponding torsion angle is shown by the C, N, and O atoms in the molecular diagram (b)). The perpendicular orientation of the benzoyl fragment found in the lowest energy conformation of the S1 state is associated with an extremely small oscillator strength, which explains low emission intensity of **ind** in solution. Conversely, the solid-state conformation is locked with the torsion angle value of ~30°, where the oscillator strength is distinctly non-zero.

12. Fitting of *in situ* spectroscopy datasets of indometacin amorphisation by milling

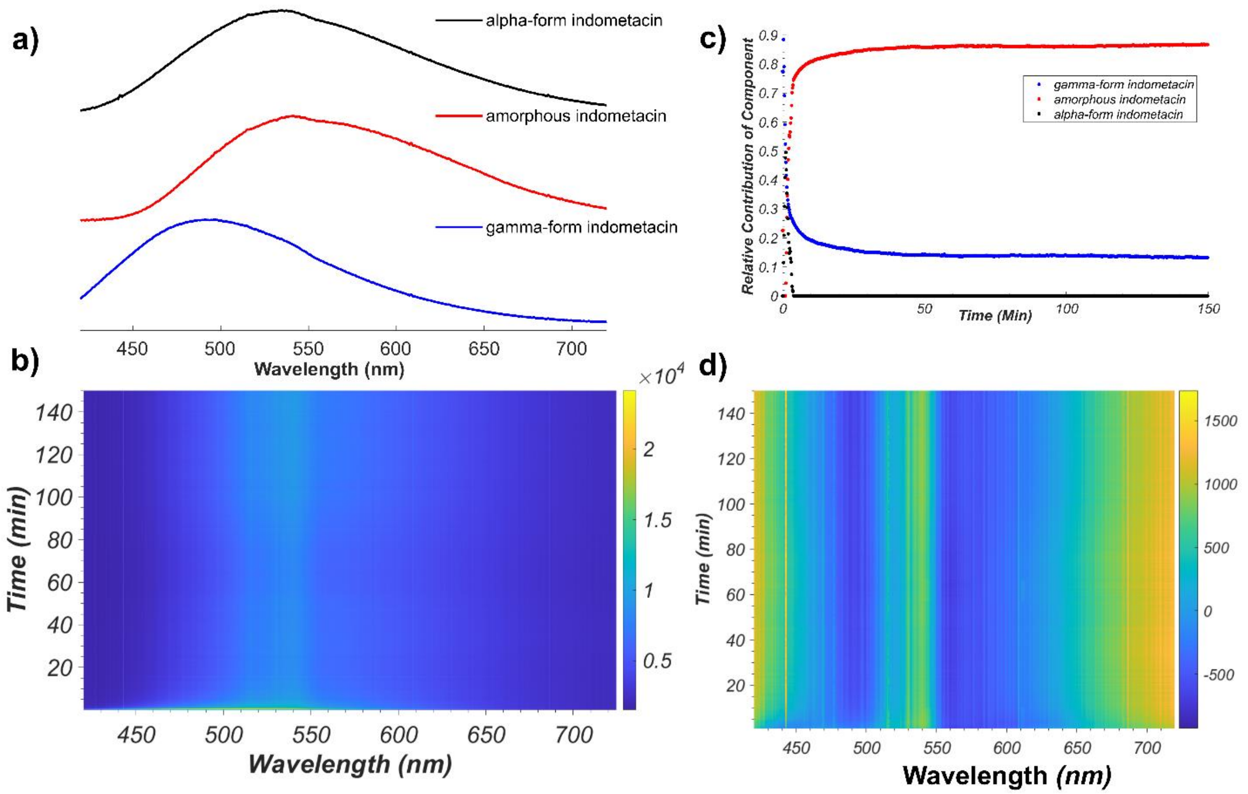

Figure S36. a) Normalised fluorescence spectra of **γ-**, **α-**, and ***am*-ind** samples synthesised *via* reported protocols.[1,2] b) Time-resolved fluorescence emission acquired during ball-milling of **γ-ind**. c) Relative amounts of **γ-**, **α-**, and ***am*-ind** estimated using non-negative least squares fitting of the *in situ* measured dataset, which fails to detect the emergence of **α-ind**. d) Time-dependent residuals after NNLS fitting.

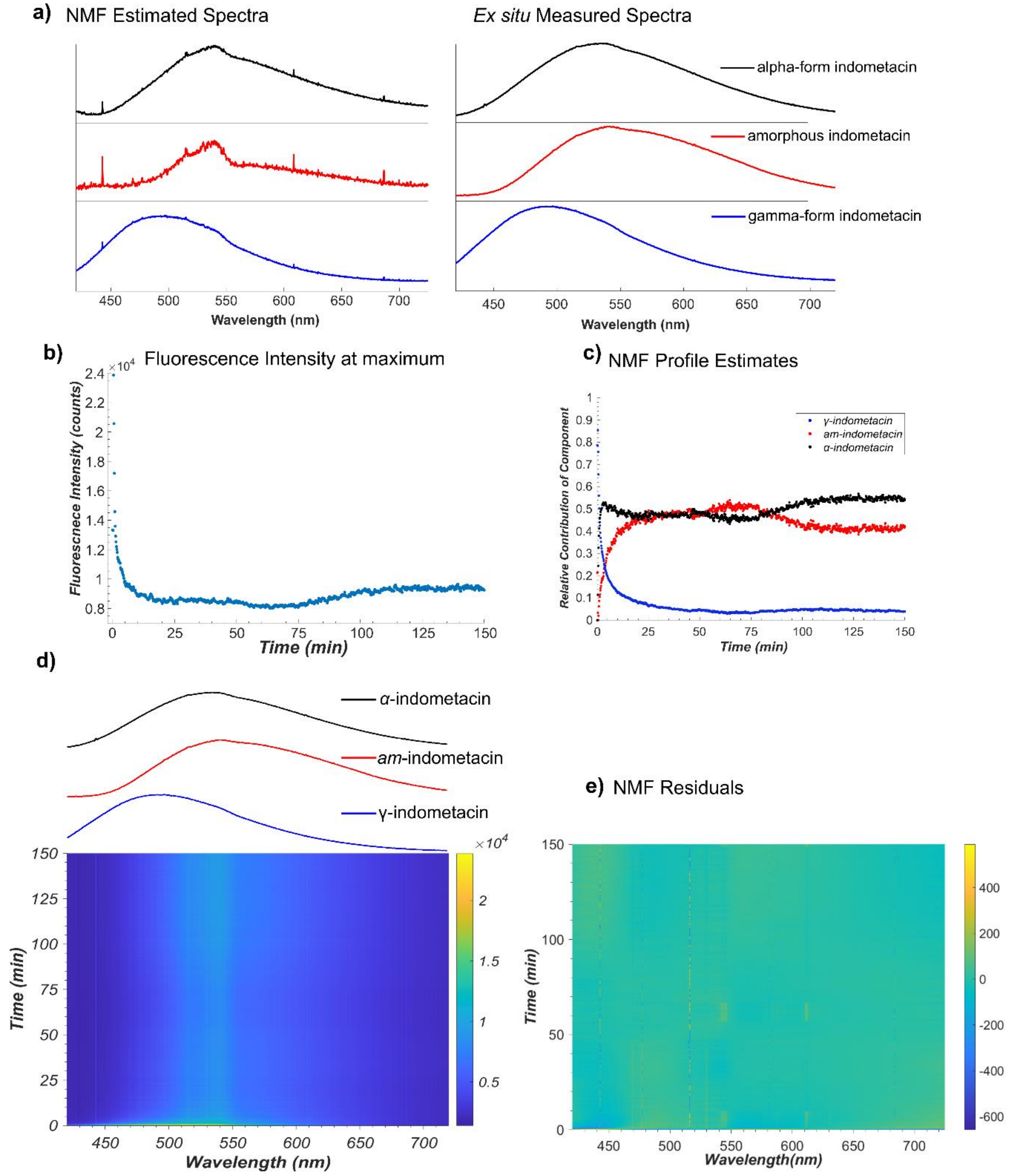

Figure S37. a) Non-negative matrix factorisation (NMF) estimated spectra (left) compared with *ex situ* normalised fluorescence emission spectra of **γ-**, **α-**, and ***am*-ind** samples synthesised *via* reported protocols.[1,2] b) Intensity of the fluorescence maximum of each real-time measured fluorescence emission spectrum. c) Reaction profile obtained from NMF fitting. d) Time resolved fluorescence emission acquired during the milling amorphisation of indometacin. e) Time-dependent residuals of the NMF.

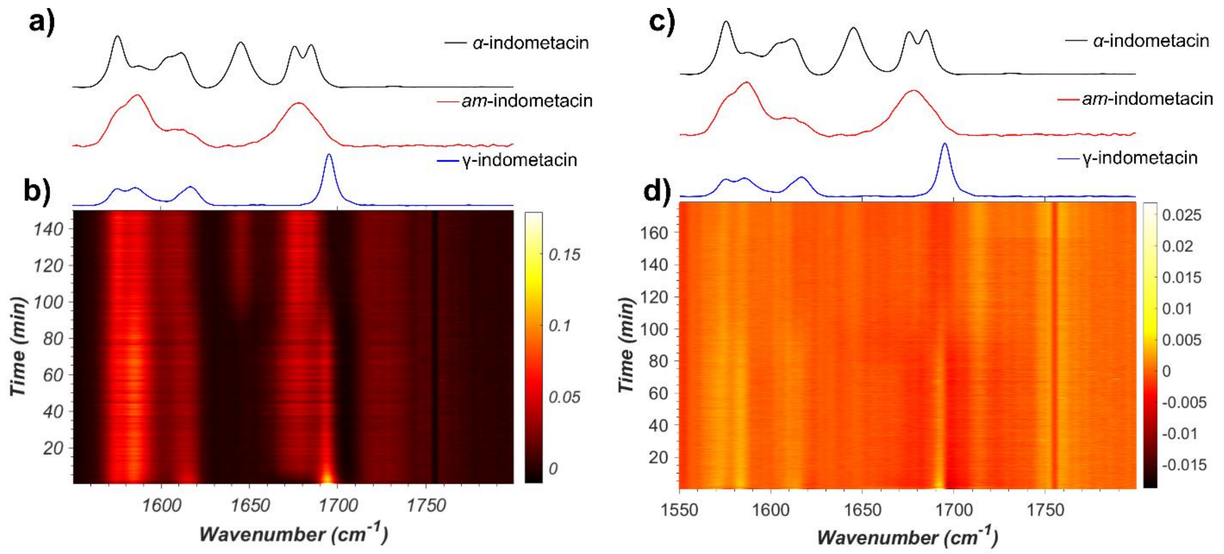

Figure S38. (a) Normalised Raman spectra of **γ-**, **α-**, and **am-ind** samples synthesised through previously reported protocols.[1, 2] (b) Time-resolved Raman spectra acquired during the milling of indometacin. (c) The same normalised spectra found in (a), and (d) time-dependent residuals after non-negative least squares fitting (NNLS) fitting.

13. Kinetic modelling of amorphisation

Amorphisation of **γ-ind:** $\alpha(t) = e^{-kt} + b$ (1) Formation of ***am*-ind:** $\alpha(t) = 1 - e^{-kt} + b$ (2)

Where the constant *b* adjusts for the baseline (minimum or maximum) of the curve in the experimental data.

Table S5. General exponential kinetic models for the amorphisation of **γ-ind** and formation of ***am*-ind**.

**Kinetic Fit for Amorphisation of γ-ind (1) from Raman Spectroscopy Measurements**	
**Coefficients (with 95% confidence bounds):**	**Goodness of fit:**
k = 0.236 (0.2282, 0.2438)	Sum of Squares Due to Error: 0.709
b = 0.1569 (0.1532, 0.1606)	R-squared: 0.9237
	Adjusted R-squared: 0.9236
	Root Mean Squared Error: 0.03773
**Kinetic Fit for Formation of am-ind (2) from Raman Spectroscopy Measurements**	
**Coefficients (with 95% confidence bounds):**	**Goodness of fit:**
k = 0.2263 (0.2189, 0.2336)	Sum of Squares Due to Error: 0.713
b = -0.1667 (-0.1705, -0.163)	R-squared: 0.9268
	Adjusted R-squared: 0.9267
	Root Mean Squared Error: 0.03784
**Kinetic Fit for Amorphisation of γ-ind (1) from Fluorescence Spectroscopy Measurements**	
**Coefficients (with 95% confidence bounds):**	**Goodness of fit:**
k = 0.5182 (0.5031, 0.5332)	Sum of Squares Due to Error: 0.5392
b = 0.05131 (0.04967, 0.05296)	R-squared: 0.8813
	Adjusted R-squared: 0.8812
	Root Mean Squared Error: 0.02449

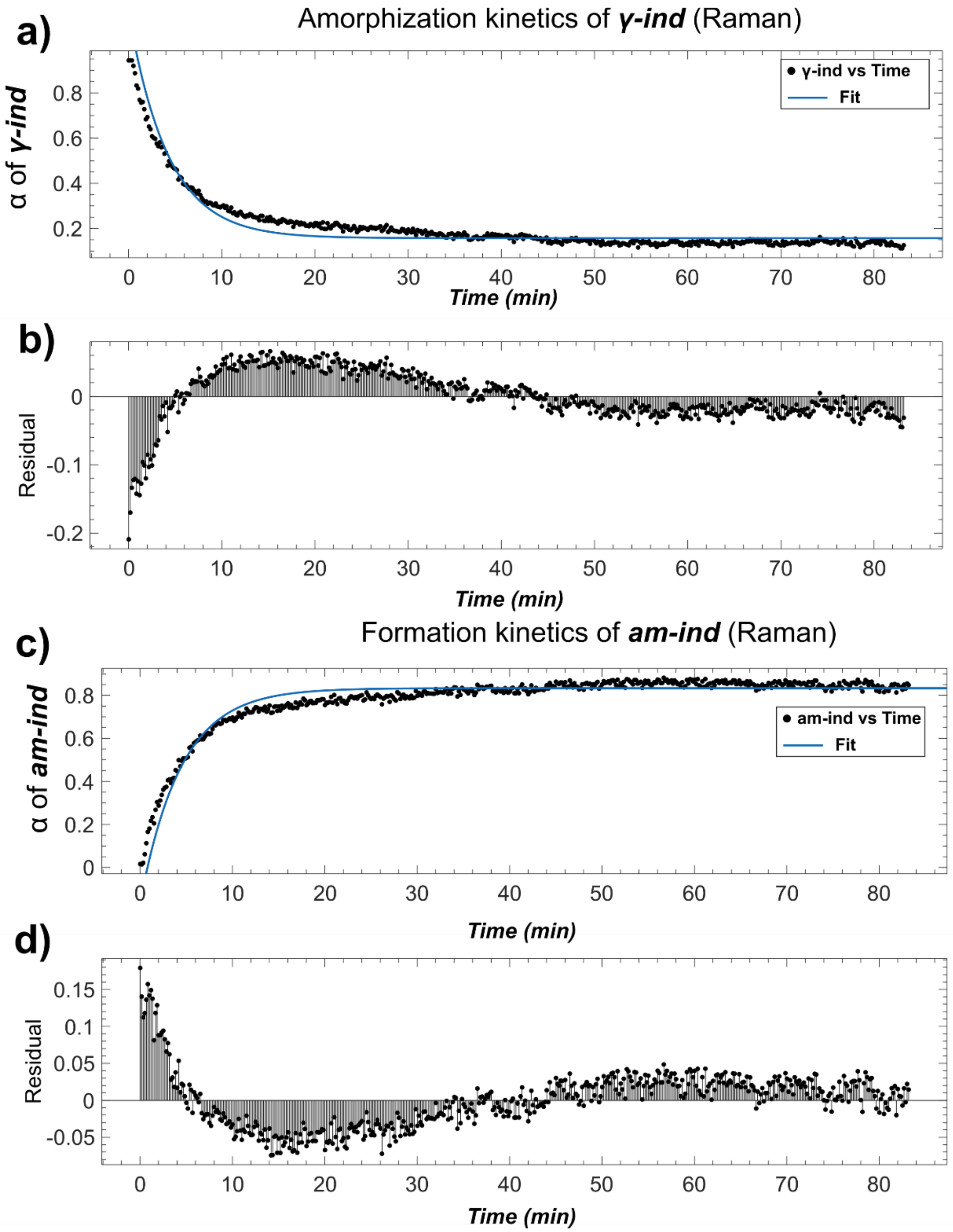

Figure S39. (a) Mol fraction of **γ-ind** (α) vs. time estimated from real-time Raman spectroscopy monitoring of the milling reaction, with fitting of the exponential kinetic model. The associated residuals after fitting are found in (b). (c) Mol fraction of ***am*-ind** (α) vs. time estimated from real-time Raman spectroscopy of the milling reaction with fit of the exponential kinetic model. The associated residuals after fitting are found in (d).

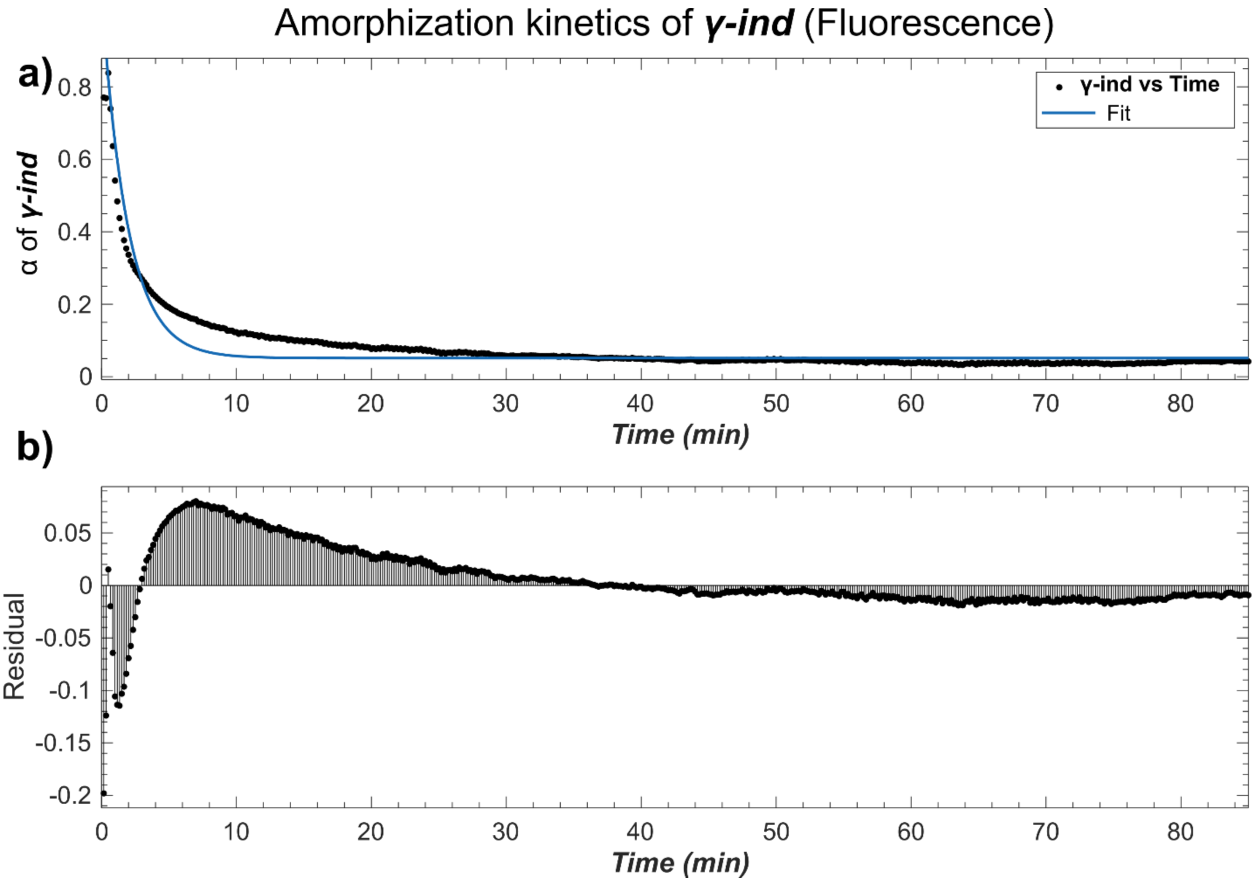

Figure S40. (a) Mol fraction of **γ-ind** (α) vs. time estimated from real-time monitoring of the milling reaction using fluorescence emission spectroscopy, including the fit of the exponential kinetic model. The associated residuals after fitting are found in (b).

14. Solid-State NMR and FTIR-ATR spectroscopy for indometacin amorphisation by ball-milling

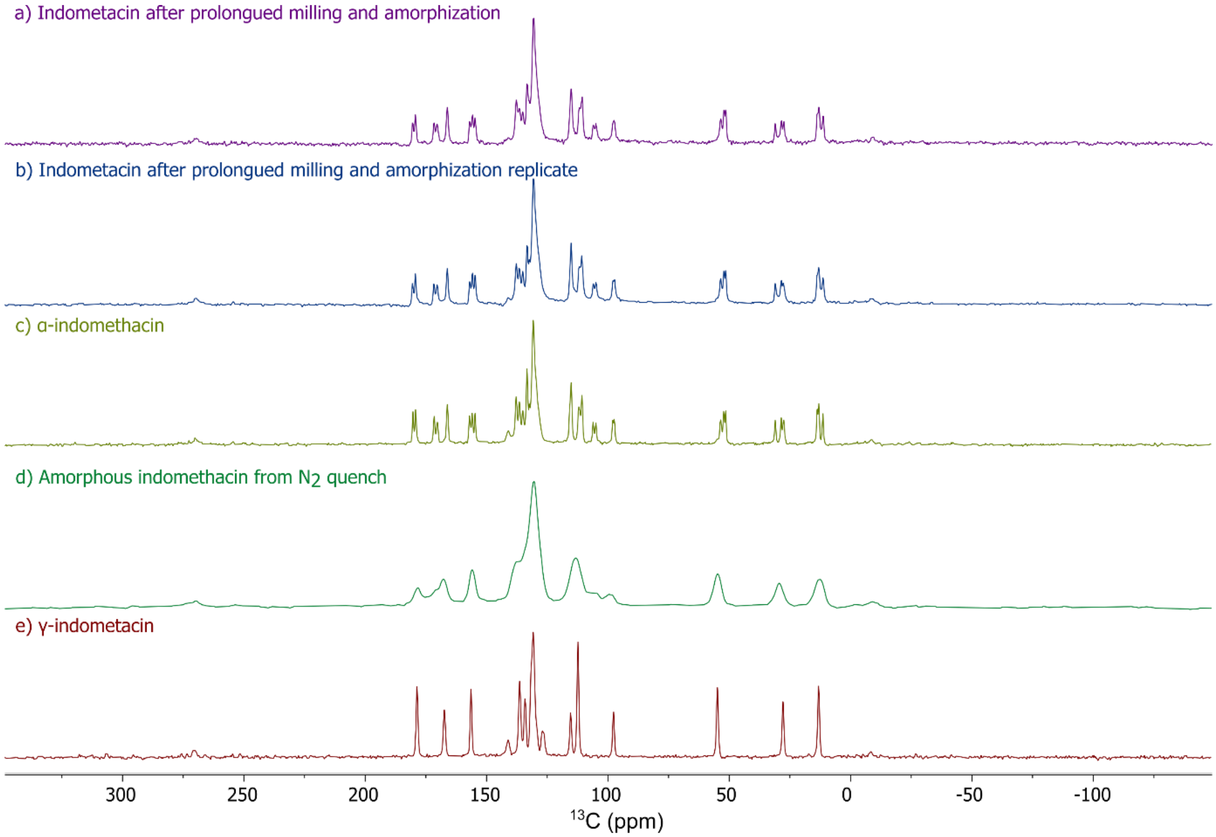

Figure S41. Comparison of ^{13}C CP-MAS-ssNMR spectra of: (a, b) **γ-ind** after continuous neat grinding, (c) solution-synthesised **α-ind**,[1] (d) **am-ind** synthesised by quenching molten **ind** in liquid nitrogen, and (e) the **γ-ind** starting material.

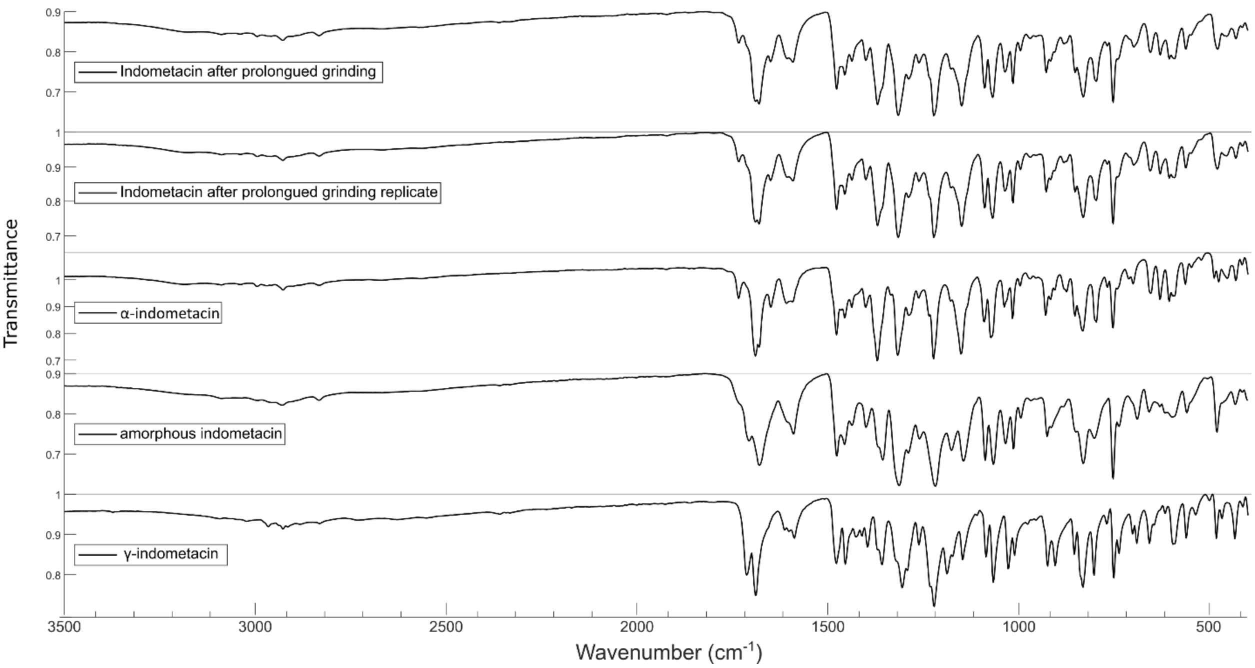

Figure S42. Comparison of FTIR-ATR spectra for (top-to-bottom): **γ-ind** after extended milling (two samples), **α-ind**,[1] ***am*-ind** *synthesised* by quenching of molten **ind** melt withliquid nitrogen, and the original **γ-ind** starting material.

References:

1. N. Kaneniwa, M. Otsuka and T. Hayashi, *Chem. Pharm. Bull.*, 1985, **33**, 3447-3455.
2. M. Yoshioka, B. C. Hancock and G. Zografi, *J. Pharm. Sci.*, 1994, **83**, 1700-1705.
